# Genetic and phenotypic architecture of human myocardial trabeculation

**DOI:** 10.1038/s44161-024-00564-3

**Published:** 2024-11-20

**Authors:** Kathryn A. McGurk, Mengyun Qiao, Sean L. Zheng, Arunashis Sau, Albert Henry, Antonio Luiz P. Ribeiro, Antônio H. Ribeiro, Fu Siong Ng, R. Thomas Lumbers, Wenjia Bai, James S. Ware, Declan P. O’Regan

**Affiliations:** 1 National Heart and Lung Institute, Imperial College London, London, UK; 2 MRC Laboratory of Medical Sciences, Imperial College London, London, UK; 3 Program in Medical and Population Genetics, The Broad Institute of MIT and Harvard, Cambridge, MA USA; 4 Department of Computing, Department of Brain Sciences and Data Science Institute, Imperial College London, London, UK; 5 Department of Cardiology, Imperial College Healthcare NHS Trust, London, UK; 6 Institute of Health Informatics, University College London, London, UK; 7 Institute of Cardiovascular Science, University College London, London, UK; 8 Department of Internal Medicine, Faculdade de Medicina, and Telehealth Center and Cardiology Service, Hospital das Clínicas, Universidade Federal de Minas Gerais, Belo Horizonte, Brazil; 9 Department of Information Technology, Uppsala University, Uppsala, Sweden; 10 Chelsea and Westminster Hospital NHS Foundation Trust, London, UK; 11 National Institute for Health Research University College London Hospitals Biomedical Research Centre, University College London, London, UK; 12 Royal Brompton and Harefield Hospitals, Guy’s and St Thomas’ NHS Foundation Trust, London, UK

**Keywords:** Cardiovascular genetics, Heart development

## Abstract

Cardiac trabeculae form a network of muscular strands that line the inner surfaces of the heart. Their development depends on multiscale morphogenetic processes and, while highly conserved across vertebrate evolution, their role in the pathophysiology of the mature heart is not fully understood. Here we report variant associations across the allele frequency spectrum for trabecular morphology in 47,803 participants of the UK Biobank using fractal dimension analysis of cardiac imaging. We identified an association between trabeculation and rare variants in 56 genes that regulate myocardial contractility and ventricular development. Genome-wide association studies identified 68 loci in pathways that regulate sarcomeric function, differentiation of the conduction system and cell fate determination. We found that trabeculation-associated variants were modifiers of cardiomyopathy phenotypes with opposing effects in hypertrophic and dilated cardiomyopathy. Together, these data provide insights into mechanisms that regulate trabecular development and plasticity, and identify a potential role in modifying monogenic disease expression.

## Abstract (web summary)

The inner surface of the heart has a meshwork of muscles called trabeculae. McGurk et al. report the genetic regulation of these complex structures across common and rare variants, revealing pathways implicated in heart development and cell fate.

## Main

The endocardial surfaces of the adult human heart are lined by a fenestrated network of muscular trabeculae that form an interface between the compact myocardium and intracardiac blood flow. Trabeculae enable nutrient and oxygen diffusion from the blood to the myocardium in early development^1^. The ventricular trabecular myocardium develops as a sponge-like network of cardiomyocytes that is critical for the development of the conduction system and ventricular chamber maturation^2,3^. Factors controlling their coordinated development are still emerging but depend on gene expression regulating cardiomyocyte polarity, cell adhesion and actin cytoskeleton dynamics, where tension heterogeneity directs the patterning of the myocardial wall during organogenesis^2,4^. The function of trabeculae in the adult heart is less well understood, but they are thought to play a role in achieving efficient cardiac performance through force transmission and modifying flow dynamics^5^.

Increased trabeculation develops in healthy individuals as a physiological and reversible phenotypic adaptation to altered loading conditions, for instance, in pregnancy and athletic training^6,7^. Excessive trabeculation is relatively benign in otherwise healthy individuals with no clinical suspicion of inherited cardiac conditions or symptoms^8^. Increased trabecular complexity is also observed in the context of heart failure, cardiomyopathy, and other circulatory and muscular disorders^3,8–10^, where it has incremental prognostic value over other clinical tests, imaging parameters and electrocardiogram (ECG) traits^11–15^. Trabecular remodeling may share mechanisms with the underlying myocardial disease^8^. While trabeculae are thought to play a pathophysiological role in the natural history of ventricular remodeling^16,17^, it is plausible that they are regulated by genetic modifiers of cardiomyopathy^3^.

Exploration of the pleiotropy between the presence of hypertrabeculation and cardiomyopathies, identification of modifiers of the natural history of trabeculation, and discovery of the full spectrum of genome-wide common and rare genetic factors with influence over the variation in trabecular morphology would aid our understanding of this complex and highly conserved phenotype.

Here, we use a deep learning approach for image segmentation and apply fractal dimension analysis as a quantitative measure of trabecular complexity^16,18–21^. We analyzed imaging in 47,803 participants of UK Biobank with genotyping and whole-exome sequencing to discover common and rare genetic variants related to adult cardiac trabeculation in the left ventricle (LV) and test potential causal relationships using genetic instruments.

## Results

### Study overview

The UK Biobank study recruited 500,000 participants aged 40–69 years in the United Kingdom between 2006 and 2010^22^. Genotyping array data and exome sequencing data were available for over 450,000 participants. A substudy recalled participants for cardiac magnetic resonance imaging (CMR)^23^, and volumetric traits were measured using quality-controlled deep learning algorithms^24–26^. Imaging was made available in two releases that formed a discovery group of 38,245 and a validation group of 9,558 participants (Supplementary Table 3).

**Fig. 1:**
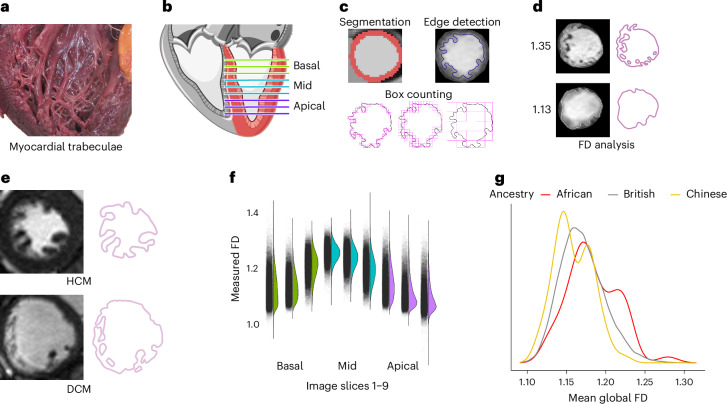
Summary of the analysis of trabeculation. **a**, Myocardial trabeculae on the endocardial surface of the human LV. **b**, CMR of the LV was acquired in the short-axis plane from base to apex. **c**, The myocardium was segmented using deep learning algorithms and edge detection used to define the boundary between the trabeculae and the blood pool. Trabecular complexity was defined by measuring the fractal dimension (FD) of this boundary using a box-counting methodology. **d**, Examples of the resulting FD output. **e**, Examples of trabecular morphology and edge detection are given for participants with HCM and DCM cardiomyopathies. The images have been reproduced with permission from the UK Biobank. **f**, The distribution of raw FD values at each level of the LV (bars represent range). **g**, The distributions of FD by ancestry. African ancestry had increased mean global FD, and Chinese ancestry had decreased mean global FD. All images show an adult human heart. Image credit: **a**, Arpatsara/Shutterstock.com; **b**, GraphicsRF/Shutterstock.com.

Trabecular morphology was quantified using edge detection of the endocardium, to derive a scale-invariant fractal dimension ratio for each slice, where a higher value indicates a greater degree of surface complexity (Fig. 1). This approach provides a measure of the complexity of the trabecular meshwork, which is a defining feature of their development and also resolves morphological variation across species and disease states^17,19^.

**Extended Data Fig. 1:**
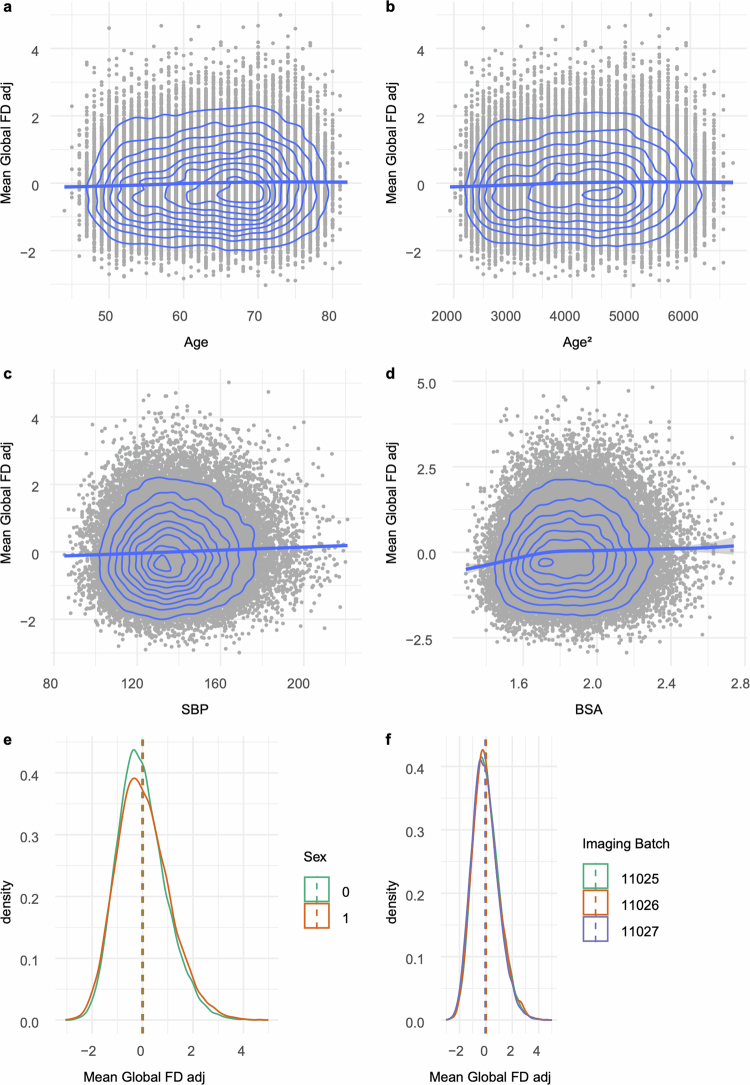
Association of covariates with mean global fractal dimension. Covariate-adjusted mean global trabeculation measured by fractal dimension analysis was compared to **a**) age at scan, **b**) age^2^, **c**) systolic blood pressure (SBP), **d**) body surface area (BSA), **e**) sex (0 = female, 1 = male), and **f**) imaging batch (three imaging centres). **a**)-**d**) are presented as scatter plots with density contours and lines of the goodness of fit (gam; formula y≈s(x)) are plotted. **e**)-**f**) are presented as overlapping density distributions with the standardised mean of 0 plotted as a vertical line for comparison.

The traits were adjusted using multiple linear regression for age at scan, age^2^, sex (not statistically significant), age:sex interaction (not statistically significant), imaging center, body surface area, systolic blood pressure (SBP), vigorous exercise and ten genetic principal components (PCs) of ancestry (statistically significant associations with PCs 1, 2, 3 and 8; Supplementary Table 1 and Extended Data Fig. 1). Comparisons were also undertaken with and without adjustment for left ventricular end-diastolic volume (LVEDV).

**Fig. 2:**
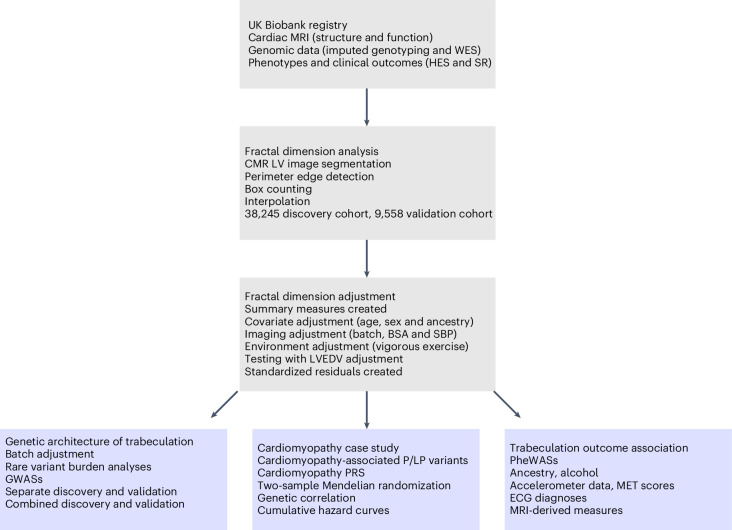
Study flowchart. A summary of the main steps in our analysis of fractal dimension and the genetic and outcome associations. MRI, magnetic resonance imaging; WES, exome sequencing; SR, self-reported; BSA, body surface area.

Trabecular traits were assessed for association with genetic, phenotypic and clinical outcome data (Fig. 2). We analyzed the cohort as separate discovery and validation cohorts as well as in combination, for rare variant burden analyses, for genome-wide association studies (GWAS) and for association with curated cardiomyopathy-associated variants. Polygenic risk scores (PRS) derived from published case–control cardiomyopathy GWAS and genetic correlation analyses were analyzed. Two-sample Mendelian randomization (MR) assessed for causality of trabeculation, cardiomyopathy and heart failure. Curated and phenome-wide association study (PheWAS) clinical outcomes were analyzed. Additional outcome and phenotypic data including hospital episode statistics (HES), self-reported questionnaire data, ancestry, alcohol, measures of physical activity, ECG diagnoses and relationships with CMR-derived measures were assessed.

### Modifiers of trabecular morphology

**Fig. 3:**
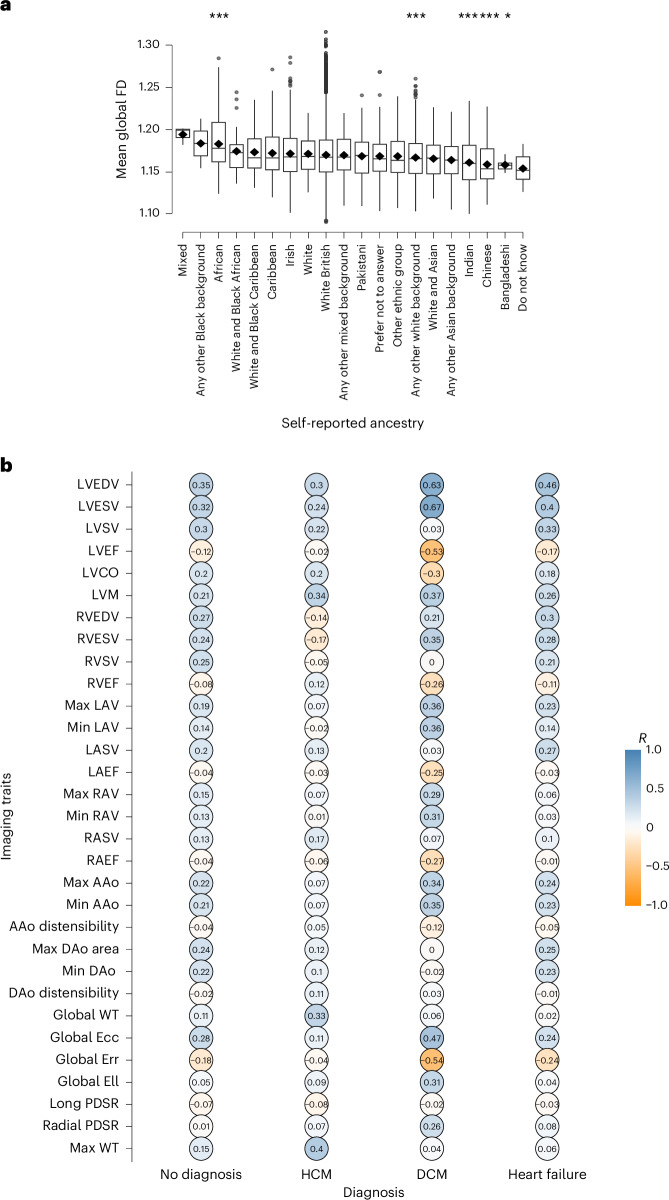
Association of trabecular morphology with ancestry and CMR-derived summary measures. The analyses were completed on 38,245 participants of the UK Biobank population. **a**, Compared with British ancestry, which dominates the UK Biobank, the mean global fractal dimension was increased for participants of African ancestry. Indian, Chinese, Bangladeshi and ‘any other white background’ self-reported ancestry had the lowest statistically significant fractal dimension (FD). Student’s two-sided *t*-test was used to compare means. The asterisks represent the *P* value. *P ≤ 0.05, ***P ≤ 0.001. For multiple comparisons, *P* ≤ 0.001 was deemed statistically significant. The presented box plots visualize the median, first and third quartiles, and the mean is presented as a diamond. **b**, The table quantifies the relationship (correlation coefficient, *R*) between mean global fractal dimension and two-dimensional summary imaging cardiac measures separately for participants with no diagnosis (*n* = 31,067), HCM (*n* = 31), DCM (*n* = 29) and heart failure (*n* = 332). EDV, end-diastolic volume; ESV, end-systolic volume; SV, stroke volume; EF, ejection fraction; CO, cardiac output; RV, right ventricle; LAV, left atrial volume; RAV; right atrial volume; AAo, ascending aortic area; DAo, descending aorta area; Ecc, circumferential strain; Err radial strain; Ell, longitudinal strain; PDSR, peak diastolic strain rate; WT, wall thickness.

African ancestry had increased mean global fractal dimension compared with white British ancestry that dominates the UK Biobank demographics (*β* = 0.28, standard error (s.e.m.) 0.07, *P* = 0.0004; Fig. 3). Indian, Chinese and Bangladeshi ancestry had the lowest mean global fractal dimension (*β* = 0.19, s.e.m. 0.08, *P* = 0.008 compared with white British ancestry). The association with African ancestry was independent of LVEDV, LV mass and body mass index.

There was no statistically significant difference in mean trabeculation (regardless of LVEDV) with obstetric history, although the hypertrabeculation observed during pregnancy is expected to be reversible^7^. Moderate or high alcohol intake (comparing the top 10% with the bottom 10% of alcohol intake (g per day (ref. ^27^))) was associated with a higher fractal dimension (*β* = 0.09, s.e.m. 0.01, *n* = 2,847 each, *P* = 0.0006; Supplementary Fig. 1), independent of LVEDV (*P* = 0.0169). Physical activity increased fractal dimension in part due to LVEDV (Supplementary Fig. 1).

**Extended Data Fig. 2:**
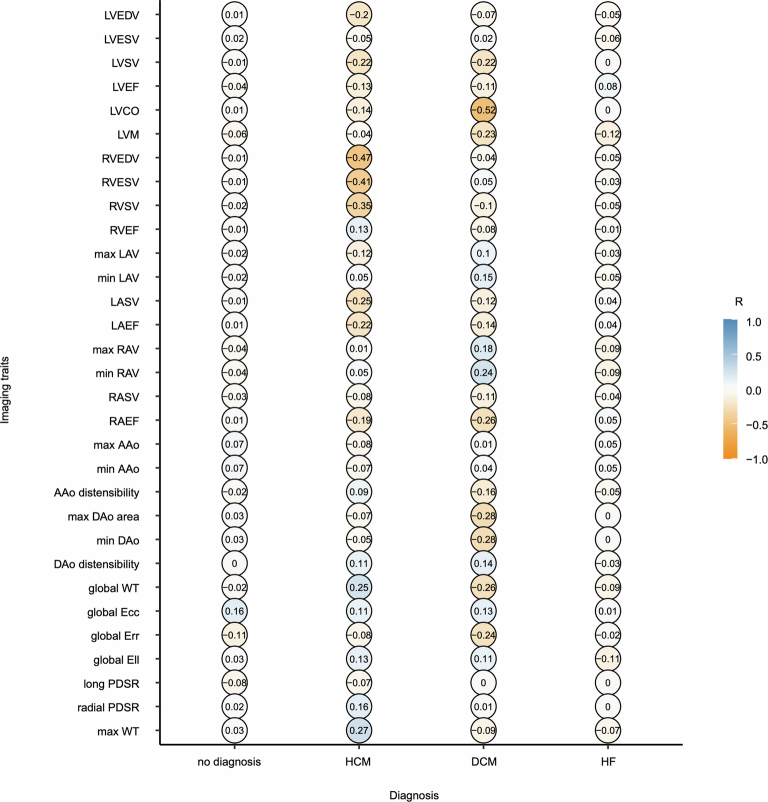
The relationship of mean global fractal dimension (FD) adjusted for left ventricular end-diastolic volume (LVEDV) with summary cardiac magnetic resonance (CMR) imaging measures in participants with cardiomyopathy or heart failure. The table quantifies the relationship (correlation coefficient, R) between mean global fractal dimension and 2D summary imaging cardiac measures separately for participants with no diagnosis (n = 31,067), hypertrophic cardiomyopathy (HCM, n = 31), dilated cardiomyopathy (DCM, n = 29), and heart failure (n = 332). LV, left ventricular; EDV, end diastolic volume; ESV, end systolic volume; SV, stroke volume; EF, ejection fraction; CO, cardiac output; RV, right ventricular; LAV, left atrial volume; RAV; right atrial volume; AAo, ascending aortic area; DAo, descending aorta area; Ecc, circumferential strain; Err radial strain; Ell, longitudinal strain; PDSR, peak diastolic strain rate.

Mean global fractal dimension had the strongest relationship with CMR measures of left ventricular volume (end-diastolic (*R* = 0.35), end-systolic (*R* = 0.32), stroke volume (*R* = 0.30)) and strain (global peak radial strain (*R* = −0.19) and global peak systolic circumferential strain (*R* = 0.28); Fig. 3 and Extended Data Fig. 2). Increased mean global fractal dimension was associated with decreased ejection fraction (*R* = −0.13), an inverse relationship that has been described for trabecular morphology previously^28^.

### PheWASs

**Fig. 4:**
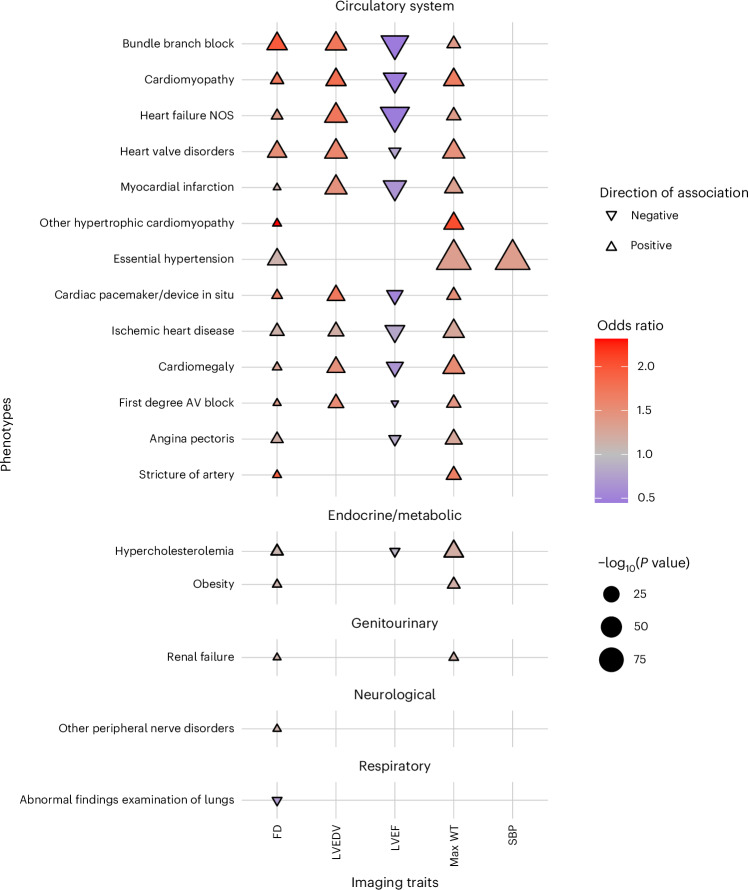
PheWAS of trabecular complexity and other CMR-derived traits. Maximum wall thickness (max WT) for HCM, LVEDV for DCM, SBP and LVEF were analyzed to assess pleiotropy between the trabecular complexity (fractal dimension, FD) and remodeling traits altered in cardiomyopathies. FD was measured at different spatial locations in the LV, and the aggregate of all results is shown. Mean global FD was associated only with cardiomyopathies, heart failure, conduction disorders and valve diseases (Extended Data Fig. 3). The analyses were completed on 38,245 participants of the UK Biobank population. Phenotypes as phecodes are described on the *y* axis, with the phecode category separating the groups, and the imaging traits are on the *x* axis. Each point denotes a statistically significant PheWAS association through linear regression with a Bonferroni correction for 1,163 analyzed phecodes. The shape and color denote the direction of effect and odds ratio. Categories and phenotypes other than the circulatory system category that did not associate with measures of trabecular morphology but were associated with the imaging measures were removed from the plot for clarity, and the most relevant traits are shown, with redundant traits shown once. NOS, not otherwise specified.

**Extended Data Fig. 3:**
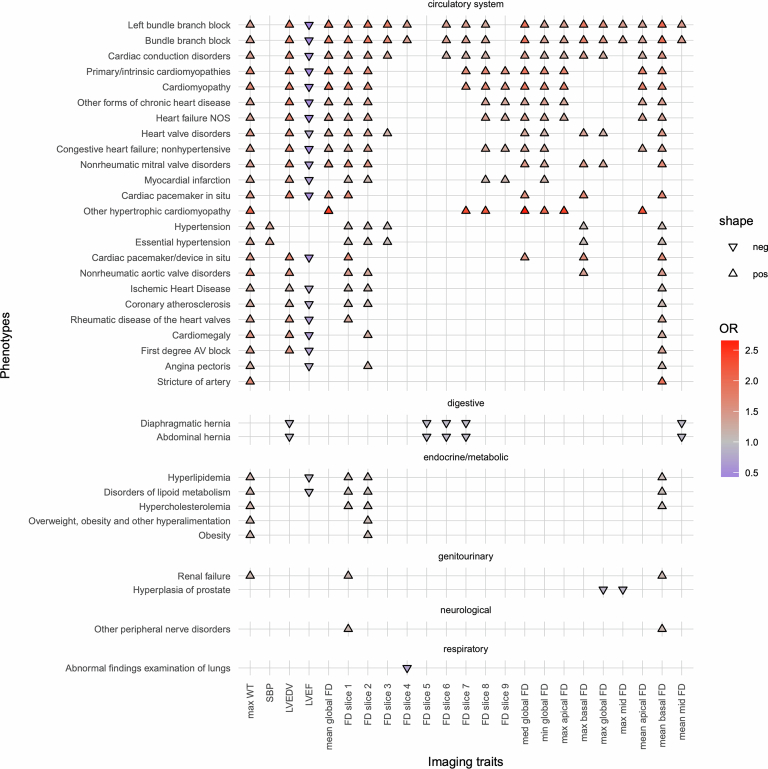
Phenome-wide association study of trabecular complexity and other CMR-derived traits. Maximum wall thickness (max WT) for hypertrophic cardiomyopathy (HCM), left ventricular end-diastolic volume (LVEDV) for dilated cardiomyopathy (DCM), systolic blood pressure (SBP), and ejection fraction (LVEF), were analyzed to assess pleiotropy between the trabecular complexity measures (FD, fractal dimension) and remodeling traits altered in cardiomyopathies. Fractal dimension was measured at different spatial locations in the left ventricle and all of the results are shown. The analyses were completed on 38,245 participants of the UK Biobank population. Phenotypes as phecodes are described on the y-axis with the phecode category separating the groups and the imaging traits are on the x-axis. Each point denotes a statistically significant PheWAS association with FD with a Bonferroni correction for 1,163 analyzed phecodes. The shape and colour denote the direction of effect (neg, negative; pos, positive) and odds ratio (OR).

**Extended Data Fig. 4:**
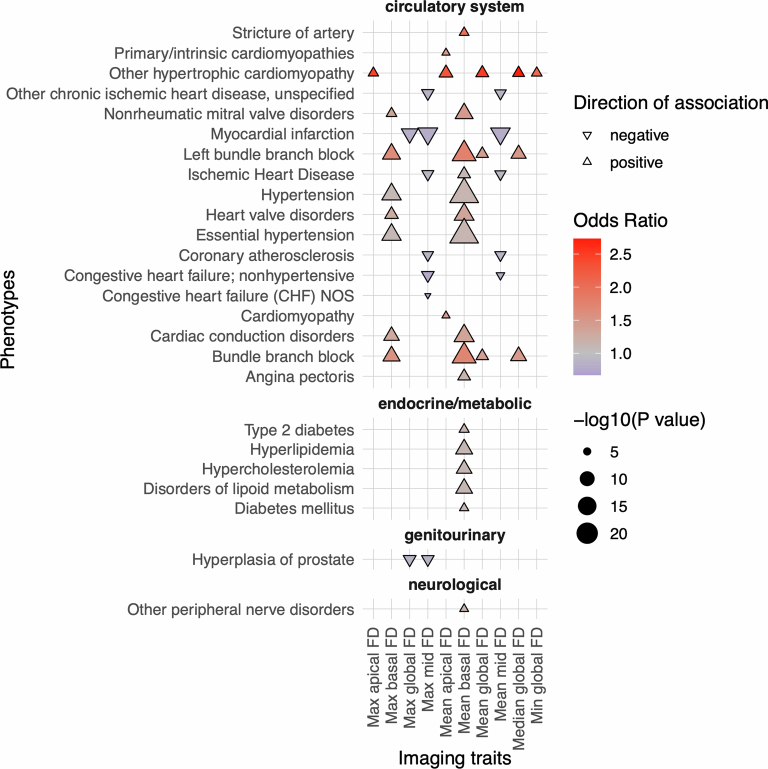
PheWAS of summary trabeculation measures when adjusted for left ventricular end diastolic volume (LVEDV). Fractal dimension was measured at different spatial locations in the left ventricle and the summary of results by region are shown. The analyses were completed on 38,245 participants of the UK Biobank population. Phenotypes as phecodes are described on the y-axis with the phecode category separating the groups and the imaging traits are on the x-axis. Each triangle denotes a statistically significant PheWAS association with a Bonferroni correction for 1,163 analyzed phecodes. The shape denotes the direction of effect and odds ratio.

PheWASs identified statistically significant associations between measures of trabecular morphology and cardiovascular disease-related clinical outcomes (Fig. 4, Extended Data Figs. 3 and 4 Supplementary Table 8). These included cardiomyopathies, heart failure, conduction disorders and valve diseases. Of note, no statistically significant association was observed with thromboembolic events or stroke. To assess for pleiotropy with current cardiomyopathy diagnostic imaging measures, we analyzed PheWAS of maximum wall thickness, SBP, LVEDV and left ventricular ejection fraction (LVEF) alongside the PheWASs of trabecular morphology. Conduction disorders, cardiomyopathies, heart failure, valve diseases and myocardial infarction were identified by all traits. Hypertension was associated with maximum wall thickness and fractal dimension. Associations with fractal dimension were identified for UK Biobank ECG diagnoses^29^ of first-degree atrioventricular (AV) block (*β* = 0.10, s.e.m. 0.03, *P* = 0.00028), left bundle branch block (*β* = 0.96, s.e.m. 0.06, *P* = 4.296 × 10^−38^), sinus bradycardia (*β* = 0.21, s.e.m. 0.02, *P* = 2.177 × 10^−26^) and atrial fibrillation (*β* = −0.39, s.e.m. 0.06, *P* = 4.307 × 10^−9^), of which left bundle branch block, sinus bradycardia and atrial fibrillation were independent of LVEDV.

### GWAS

The single nucleotide polymorphism (SNP)-based heritability was estimated for each of the trabecular morphology measures (Supplementary Table 3). Mean global fractal dimension had the highest estimated heritability (*h*^2^ = 0.43). The most basal and apical slices had heritability estimates of approximately 0.2, while slices in the mid-region of the LV had the highest estimated heritability (*h*^2^ ≈ 0.4).

**Fig. 5:**
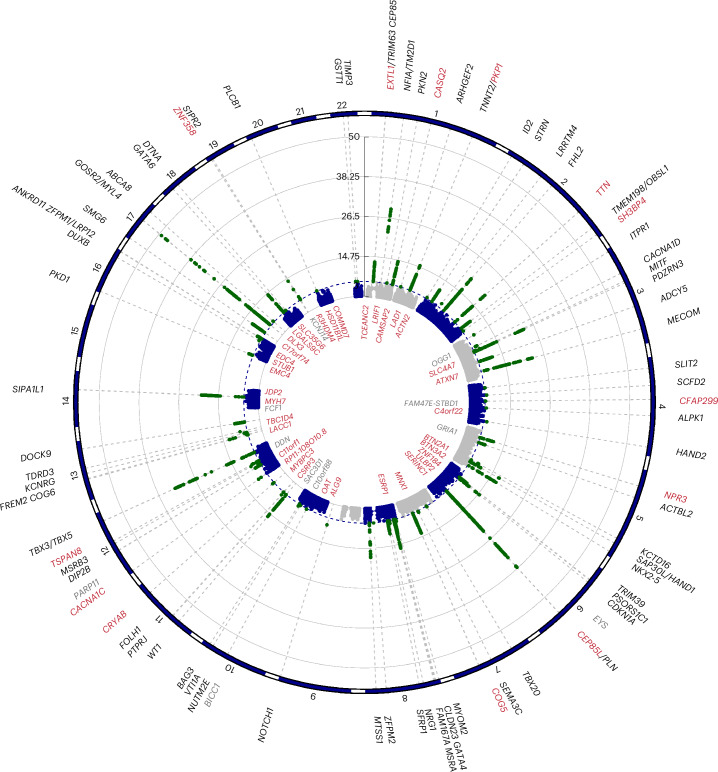
Circular Manhattan plot for genetic loci associated with trabeculation. Common and rare variant genetic analyses for trabecular complexity assessed with fractal dimension analysis. The prioritized gene is noted for the statistically significant loci identified through combined discovery and validation of common variant GWAS on the outside of a meta-Manhattan plot of the minimum *P* value for each SNP across measures of trabeculation. The RVAS identified genes with a burden of PAVs that are associated with trabecular morphology (depicted inside the circle). Genes in red on the outside circle denote a gene identified through both GWAS and RVAS. Genes in gray were identified only in the discovery analysis and not in the full analysis of combined discovery and validation cohorts. Created using CMplot in R. The GWAS results were statistically significant if *P* < 5 × 10^−8^. The RVAS results were statistically significant after adjusting for the number of genes analyzed.

We identified 68 loci from GWASs of 38,012 European participants and replicated previous studies^17^ (Fig. 5 and Supplementary Tables 4, 5 and 9). We used LocusZoom, expression quantitative trait loci and transcriptome-wide association study (TWAS) to prioritize genes at each locus. The identified associations include loci of definitive evidence cardiomyopathy-associated genes (*TNNT2*, *TTN*, *PLN* and *BAG3*) and colocalize with recent hypertrophic cardiomyopathy (HCM) (for example, *TBX3*, *STRN* and *MTSS1* (ref. ^30^)), dilated cardiomyopathy (DCM) (for example, *PKD1*, *STRN*, *MITF* and *CDKN1A*^31^) and heart failure GWAS^32^. We identified further genes that regulate the development of trabeculation (*NKX2-5*, *TBX20*, *HAND2*, *NRG1*, *NOTCH1* and *DTNA*) and those with evidence of involvement in cardiomyopathies (*CRYAB* and *RIT1* (*ARHGEF2*)). We validated 13 loci through rare variant association studies (RVASs; *EXTL1*, *CASQ2*, *CEP85L*, *COG5*, *TSPAN8*, *TTN*, *SH3BP4*, *CFAP299*, *CRYAB*, *CACNA1C*, *PKP1* (*TNNT2*), *NPR3* and *ZNF358*). Statistically significant Gene Ontology (GO) resource enrichment analysis showed the strongest relationship with cardiac myofibril and sarcomeric cellular components, electrical conduction His cell differentiation, ventricle formation and muscle cell fate commitment (Supplementary Table 13).

We found an overlap with loci identified here and those identified from GWAS of QRS duration^33^: calcium handling genes (*CASQ2*, *PLN* and *CACNA1D*), genes of transcription factors (*TBX3*, *HAND1* and *NFIA*), cyclin-dependent kinase inhibitors (*CDKN1A*) and others (*SIPA1L1*, *GOSR2* and *STRN*). The identification of the loci of *TTN*, *NKX2-5*, *MTSS1*, *TNNT2* and *CACNA1C* suggests a relationship with trabeculation in ventricular depolarization and repolarization^34^. Many genes identified here have been found to influence ECG traits (*TRIM63*, *DES*, *NPR3*, *NKX2-5*, *CDKN1A*, *SFRP1*, *WT1*, *ZFPM1*, *GATA6*, *NFIA*, *TNNT2*, *TTN*, *PDZRN3*, *HAND1*, *MTSS1*, *DEFB136*, *TBX3*, *GOSR2/MYH6*, *ZNF358*, *FLRT2* and *HAND2*)^34^. *CACNA1D* has moderate evidence for causing autosomal recessive sinoatrial node dysfunction and deafness.

The association with the genes *NKX2-5*, *NOTCH1*, *GATA4*, *GATA6* and *HAND1* links trabeculation to congenital heart disease (CHD). *NKX2-5*, *NOTCH1* and *GATA4* have high confidence for nonsyndromic CHD, atrial septal defect (*NKX2-5* and *GATA4*), aortic valve stenosis and tetralogy of fallot (*NOTCH1*). *NKX2-5* when suppressed causes excessive trabeculation in models^8^, and it is identified in carriers with left ventricular noncompaction alongside DCM or conduction disease^35^. GATA6 is a transcription factor with a role during heart development and has been linked to several CHD phenotypes previously^36^, and *HAND1* has moderate evidence for CHD^37^. *PKD1* is associated with polycystic kidney disease, a syndromic CHD^37^.

For sensitivity analyses, we identified 32 loci from GWASs of 30,419 participants with validation in 7,593 participants (Fig. 5 and Supplementary Tables 4 and 5), removed individuals who had a diagnosis of any cardiomyopathy or heart failure (all variants replicated except for the loci at *SFRP1*, *NUTM2E*, *SCFD2* and *EYS*), adjusted for LVEDV (loci at *MITF*, *PSORS1C1*, *EYS* and *BICC1* were less statistically significant (*P* ≥ 5 × 10^−8^; Supplementary Fig. 2)) and inverse rank normalized the data (Supplementary Tables 5 and 9).

### RVASs

We identified 56 genes in which rare protein-altering variants (PAVs) were associated with trabecular morphology (Fig. 5 and Supplementary Tables 7 and 10). This included genes involved in inherited cardiac conditions, involved in calcium signaling, implicated in neurodevelopment and that regulate trabeculation.

Trabeculation was influenced by genes with roles in cardiac function and disease (*TTN*, *MYBPC3*, *MYH7*, *CRYAB*, *CACNA1C*, *CASQ2* and *PLN*). *TTN* encodes a large, abundant protein of striated muscle that has a key role in DCM. Loss-of-function variants increased trabecular morphology with the strongest effects toward the apex. Myosin-binding protein C3 (*MYBPC3*) encodes the cardiac isoform of myosin-binding protein C. PAVs in *MYBPC3* are the most common genetic cause of HCM^38^. Missense variants in cardiac myosin heavy chain β (*MYH7*) have definitive evidence of causation of HCM or DCM (via distinct mechanisms) and increased trabecular complexity. Crystallin alpha B (*CRYAB*) is an aggregating chaperone that is mainly expressed in the heart and has definitive evidence for causing alpha-B crystallinopathy^38^, where left ventricular hypertrophy is observed with overt syndromic features. Missense variants in *MYBPC3*, *MYH7* and *CRYAB* increased apical trabecular complexity. Calcium voltage-gated channel subunit alpha 1C (*CACNA1C*) is involved in cellular calcium influx after depolarization. The gene has definitive evidence for Timothy syndrome, which is a multiorgan disorder that includes cardiovascular malformation and fatal arrhythmias. Missense variants in the gene decreased mid-trabecular complexity. Calsequestrin 2 (*CASQ2*) encodes a calcium-binding protein that stores calcium for muscle function. It is highly expressed in the heart and arteries and has definitive evidence for causing catecholaminergic polymorphic ventricular tachycardia. Missense variants in the gene associated with decreased apical trabecular complexity. Centrosomal protein 85 like (*CEP85L*) has broad tissue expression and has been linked to cancer and lissencephaly. The single exon gene phospholamban (*PLN*) sits within the *CEP85L* locus. *PLN* has the highest expression in the heart and arteries and has definitive evidence for HCM. Missense variants in the *CEP85L* region decreased apical trabecular complexity.

### Genetic modifiers of trabeculation in cardiomyopathy

Hypertrabeculation occurs in patients with HCM and DCM^17,25,39^, although the morphology of trabeculae in these cardiomyopathies may reflect the underlying disease and pattern of remodeling. In the UK Biobank, fractal dimension was increased in participants diagnosed with hypertrophic (*β* = 0.95, s.e.m. 0.17, *P* = 8.6 × 10^−5^) and dilated (*β* = 0.74, s.e.m. 0.16, *P* = 0.01) cardiomyopathies and CHD (*β* = 0.37, s.e.m. 0.09, *P* = 0.002). The relationship between trabeculation and HCM was independent of LVEDV and wall thickness but was related to the degree of ventricular dilatation in participants with DCM (Fig. 3 and Extended Data Fig. 2). Furthermore, adjusting mean global fractal dimension for LVEDV, maximum wall thickness and LVEF showed that trabeculation remained significantly increased in HCM (*β* = 0.74, *P* = 0.0003), in participants with aggregated International Classification of Diseases (ICD) codes for AV and left bundle-branch block (ICD codes I44.*; *β* = 0.13, *P* = 0.008) and an ECG diagnosis of left bundle-branch block (*β* = 0.56, *P* = 1.69 × 10^−15^).

**Extended Data Fig. 5:**
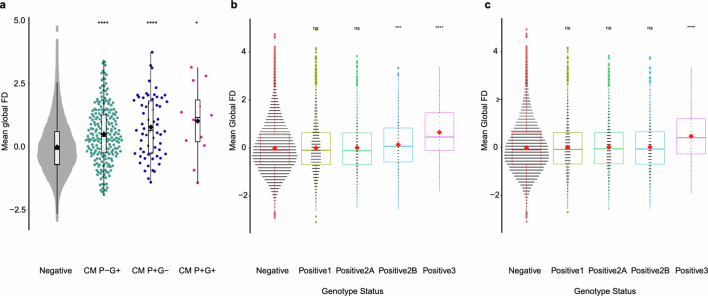
Trabeculation and variant pathogenicity. Student’s two-sided t-test was used to compare the group means. The lower and upper hinges in the box plot correspond to the 25th and 75th percentiles (interquartile range (IQR)), respectively. The horizontal line in the box plot indicates the median and the diamond indicates the mean. The lower and upper whiskers extend from the hinge to the smallest and largest values no further than 1.5 × the IQR, respectively. Each dot is one individual. *, *P* < 0.05; **, *P* < 0.01; ***, *P* < 0.001; ****, *P* < 0.0001, compared to the negative reference group. For multiple comparisons, a *P* ≤ 0.001 was deemed statistically significant. CM, cardiomyopathy. **a)** Carriers of cardiomyopathy-associated pathogenic or likely pathogenic variants (G+) with or without a diagnosis of disease (phenotype; P+/-) had increased mean trabeculation compared to genotype negatives. P-G+, phenotype negative, genotype positive; P+G-, phenotype positive, genotype negative; P+G+, phenotype positive, genotype positive; Negative, genotype and phenotype negative. There were 23,936 genotype-negative participants, 222 participants with a P/LP variant without a diagnosis of cardiomyopathy, 63 participants with disease and no variant in definitive-evidence genes, and 12 participants with a variant and diagnosis of disease. These independent groups of individuals for **b)** HCM and **c)** DCM, were separated by carrier status. For HCM-associated variants, there were 28,006 genotype-negative participants (Negative), 6,040 participants with variants in syndromic genes (Positive1), 1,330 participants with common variants (AF < 0.01 & > 0.00004) in definitive-evidence genes (Positive2A), 680 participants with rare indeterminant (VUS) variants in definitive-evidence genes (Positive2B - statistically significant), 72 carriers of P/LP variants (Positive3 - statistically significant). For DCM-associated variants, there were 28,109 genotype-negative participants (Negative), 3,379 participants with variants in syndromic genes (Positive1), 2,710 participants with common variants (AF < 0.01*&* > 0.000084) in definitive-evidence genes (Positive2A), 1,837 participants with rare indeterminant variants in definitive-evidence genes (Positive2B), 162 carriers of P/LP variants (Positive 3 - statistically significant).

**Extended Data Fig. 6:**
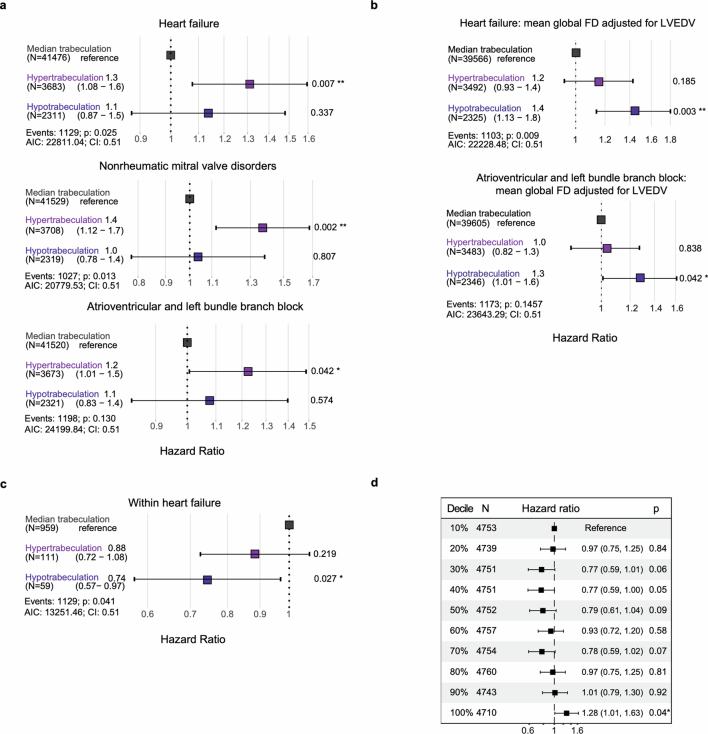
Outcome associations. The analyses were completed on 47,803 participants of the UK Biobank population and assessed from years since imaging for a diagnosis of a cardiac condition or all-cause death, with anyone diagnosed before imaging excluded. Trabeculation was separated into three groups: hyper-, median, and hypotrabeculation where < 1.5 standard deviation (SD) is hypotrabeculation and > 1.5 SD is hypertrabeculation. The dashed verticle line represents the comparative reference group (Midtrabeculation). Hazard ratios and 95% confidence intervals are presented. **a**, The hazard ratio for heart failure, for mitral valve disorders, and bundle branch block, showed increased risk with hypertrabeculation. **b**, The hazard ratio with the adjustment for left ventricular end diastolic volume (LVEDV) (and removal of the association of trabeculation with dilated cardiomyopathy (DCM)) showed that hypotrabeculation was associated with the risk of non-DCM heart failure. **c**, The hazard ratio for assessments with participants diagnosed with heart failure only showed that heart failure risk increased with hypotrabeculation. Of those with heart failure or death, participants with hypotrabeculation were diagnosed or died nearly a year later on average. **d**, The mean coxfit linear predictors for heart failure were plotted for trabeculation by decile. Confidence intervals and log-rank *P* values are depicted. CI, concordance index.

**Extended Data Fig. 7:**
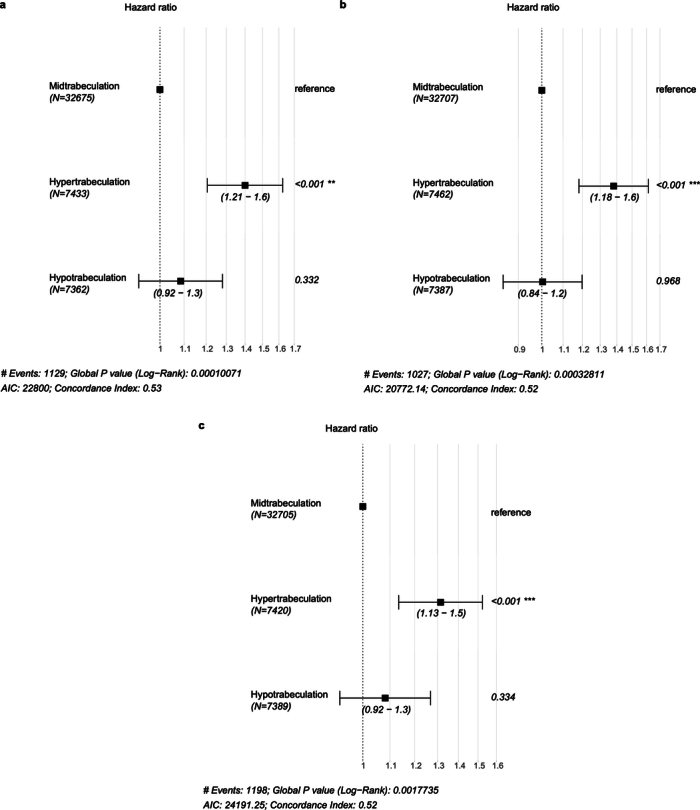
Sensitivity analysis of the association of trabeculation outside 1.5 standard deviations (SD) with clinical outcomes. The plots show the same analysis completed for 1.5 SD before by 1 SD for **a**, heart failure (hypertrabeculation *P* = 9.83 x 10^−6^), **b**, mitral valve disease (hypertrabeculation *P* = 4.96 x 10^−5^), and **c**, bundle branch block (hypertrabeculation *P* = 0.00027). The mean coxfit linear predictors were plotted for trabeculation by group. Confidence intervals and log-rank *P* values are depicted.

**Extended Data Fig. 8:**
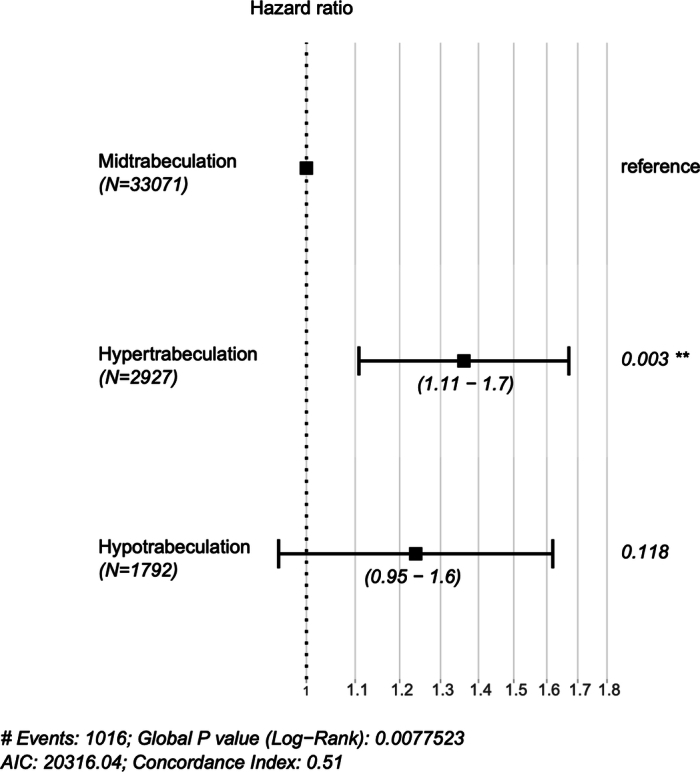
Association of fractal dimension and heart failure risk in participants with non-dilated non-hypertrophied ventricles. The analyses were completed on 47,803 participants of the UK Biobank population and assessed from years since imaging for a diagnosis of a cardiac condition or all-cause death, with anyone diagnosed before imaging excluded. Trabeculation was separated into three groups: hyper-, median, and hypotrabeculation where < 1.5 standard deviation (SD) is hypotrabeculation and > 1.5 SD is hypertrabeculation. The dashed vertical line represents the comparative reference group (Midtrabeculation). Hazard ratios and 95% confidence intervals (CIs) are presented.

About 30% of cardiomyopathy cases are attributable to a rare variant in a cardiomyopathy-associated gene, many of which encode components of the sarcomere^40^. After excluding individuals with a diagnosis of cardiomyopathy, UK Biobank participants carrying either a HCM or DCM-associated pathogenic or likely pathogenic variant had increased fractal dimension (compared with participants without such a variant; Extended Data Fig. 5). Participants with a diagnosis of cardiomyopathy also had increased fractal dimension, irrespective of the presence/absence of a potentially causative sarcomere variant. The presence of hypertrabeculation on imaging was a risk factor for a subsequent diagnosis of heart failure (hazard ratio (HR) 1.3, 95% confidence interval (CI) 1.08–1.6), mitral valve disorders (HR 1.4, 95% CI 1.12–1.70) and bundle branch block (HR 1.2, 95% CI 1.01–1.50; Extended Data Figs. 6 and 7 and Supplementary Figs. 11 and 12). Hyper- and hypotrabeculation were described as outside 1.5 standard deviation (s.d.) of the mean. Sensitivity analyses have been completed using 1 s.d., 2 s.d. and deciles (Extended Data Fig. 7 and Supplementary Figs. 11 and 12). Of 81 people with cardiomyopathy reported after imaging, 25% (*n* = 20) had hypertrabeculation and none had hypotrabeculation. A less trabeculated ventricle was a risk factor for heart failure when adjusted for LVEDV (Extended Data Fig. 6). Increased trabeculation remained significantly increased in participants with a diagnosis of cardiomyopathy when individuals with phenotypic HCM (maximum wall thickness ≥15 mm in the absence of a diagnosis of hypertension or valve disease) or DCM (LVEDV larger than two s.d. by sex^41^ and LVEF <50%) on imaging were excluded (HCM, *β* = 0.95, *P* = 8.27 × 10^−6^; DCM, *β* = 0.74, *P* = 0.001). Increased fractal dimension in participants with nondilated nonhypertrophied ventricles have a HR of 1.36 (95% CI 1.11–1.67, *P* = 0.003) for future heart failure diagnoses (Extended Data Fig. 8).

PRS for HCM derived from case–control common variant GWAS showed an inverse relationship with fractal dimension in UK Biobank (comparing the lowest 10% of the PRS with the other 90%: *β* = −0.08, s.e.m. 0.02, *n* = 3,039 versus 27,350, *P* = 2.3 × 10^−5^; and comparing the top 10% of the PRS with the bottom 10%: *β* = −0.12, s.e.m. 0.01, *n* = 3,039 each, *P* = 3.6 × 10^−6^), while the opposite relationship was identified with the DCM PRS (top 10% of the PRS to the other 90%: *β* = 0.06, s.e.m. 0.02, *n* = 3,042 versus 27,377, *P* = 1.0 × 10^−3^; and top 10% of the PRS to the bottom 10%: *β* = 0.11, s.e.m. 0.01, *n* = 3,042 each, *P* = 3.8 × 10^−5^). The strongest genetic covariance (*C*_g_ = 0.15–0.16) and correlation (*r*_g_ = 0.40–0.43) was between trabecular morphology and LVEDV (Supplementary Table 2). A negative correlation was observed with maximum wall thickness (*r*_g_ = −0.10 to −0.05; Supplementary Table 2).

**Fig. 6:**
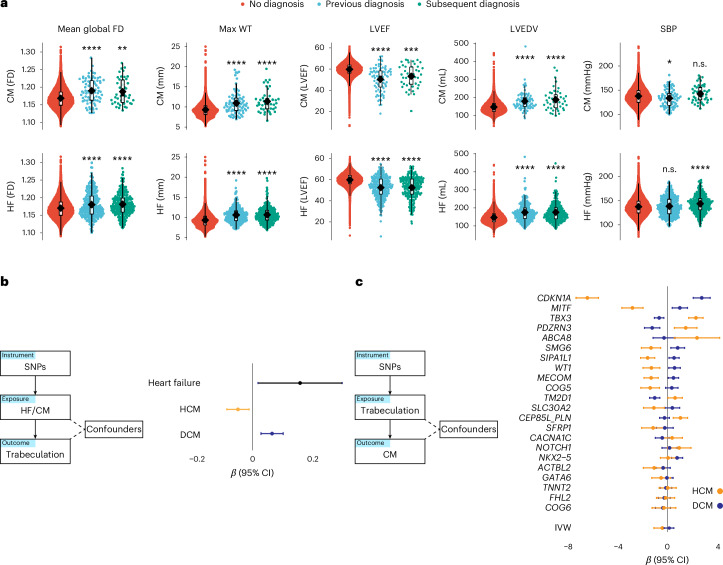
Relationship between trabecular complexity and cardiomyopathy. Increased fractal dimension (FD) may occur early in the natural history of cardiomyopathies (CMs) and heart failure (HF). **a**, Imaging biomarkers (LVEDV, ejection fraction (LVEF), maximum wall thickness (max WT), SBP and mean global fractal dimension) for three groups of participants depending on CM or HF diagnosis status; as (i) no diagnosis, (ii) diagnosis recorded previous to imaging or (iii) diagnosis recorded after imaging. Analyses were performed on 38,245 participants of the UK Biobank. Student’s two-sided *t*-test was used to compare the group means to individuals with no diagnosis. The lower and upper hinges in the box plot correspond to the 25th and 75th percentiles (interquartile range, IQR), respectively. The horizontal line in the box plot indicates the median. The lower and upper whiskers extend from the hinge to the smallest and largest values no further than 1.5× the IQR, respectively. Each dot is one individual. The asterisks represent the *P* value. n.s., not statistically significant, *P* > 0.05; *P ≤ 0.05, **P ≤ 0.01, ***P ≤ 0.001, ****P ≤ 0.0001. For multiple comparisons, *P* ≤ 0.001 was deemed statistically significant. **b**, MR genetic determination model of CM and HF genetic instruments as exposures for trabeculation outcome. Common genetic loci influencing the variability of cardiac function in an additive fashion may be deferentially involved depending on the CM substrate. Common genetic variants associated with DCM increased trabecular complexity and HF risk. Common genetic variants associated with HCM had an inverse relationship with trabeculation. Two-sample MR was undertaken with genetically determined mean global FD (using summary statistics from the GWAS of 38,245 European participants) and genetically determined DCM, HCM and HF (from GWAS summary statistics of published data). Presented are the inverse variance weighted (IVW) estimates. **c**, MR genetic determination model of trabeculation genetic instruments (from GWAS of 38,245 participants) as exposures for CM outcomes was not statistically significant (IVW). The single SNP funnel plot for trabeculation genetic instruments as exposures for cardiomyopathies shows that trabeculation-associated variants have opposing directions of effect for DCM or HCM. The plot is ordered by the delta of the *β* values; *β* and 95% CIs are presented with a vertical line at *β* = 0. Four variants were removed that were not present in the HCM summary statistics so GWAS *β* values could not be compared with DCM.

**Extended Data Fig. 9:**
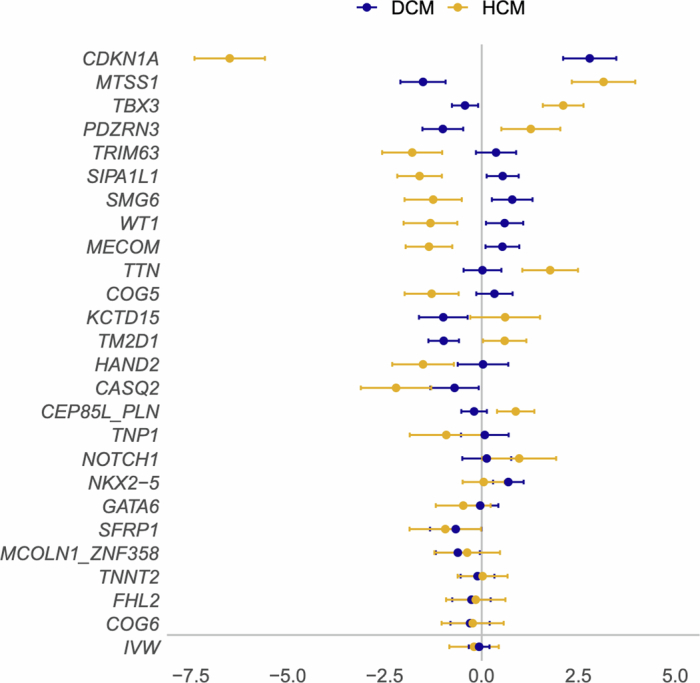
Relationship between trabecular complexity and cardiomyopathy. Mendelian randomization genetic determination model of trabeculation genetic instruments adjusted for left ventricular end diastolic volume (LVEDV; from GWAS of 38,245 European participants) as exposures for cardiomyopathy outcomes was not statistically significant (inverse variance weighted; IVW). The single SNP funnel plot for trabeculation genetic instruments as exposures for cardiomyopathies shows that trabeculation-associated variants have opposing directions of effect for dilated cardiomyopathy (DCM) or hypertrophic cardiomyopathy (HCM). The plot is ordered by the delta of the betas.

**Extended Data Fig. 10:**
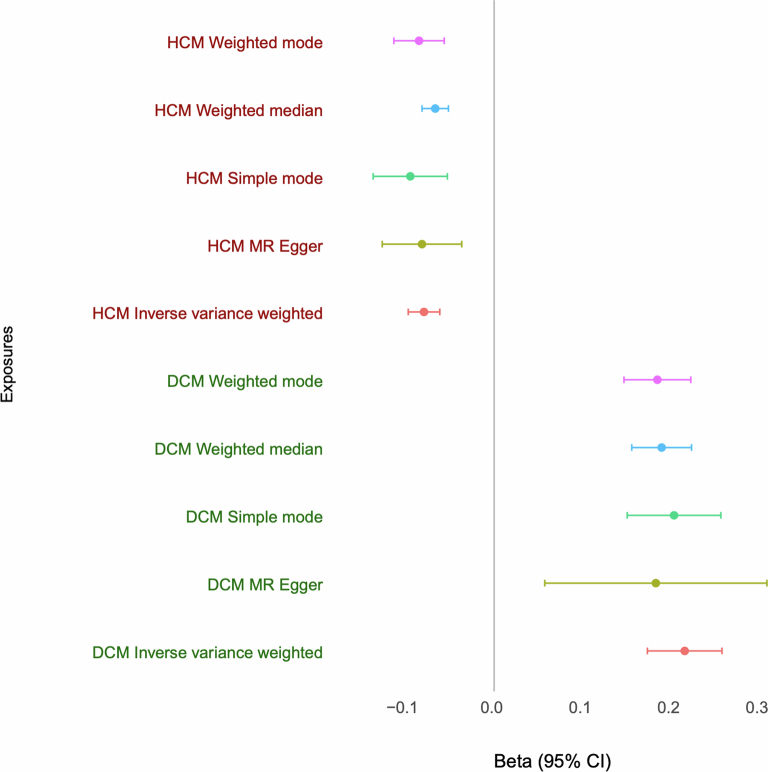
Mendelian randomization analysis of dilated cardiomyopathy (DCM) and hypertrophic cardiomyopathy (HCM) as exposures on heart failure outcomes. The plot shows summary information on the analyses, performed as per the TwoSampleMR R package using publicly available GWAS results (see Methods). The vertical line represents *β* = 0. Betas and 95% confidence intervals (CIs) are presented.

To assess causality, we used two-sample MR for association testing of genetically predicted levels of an exposure on an outcome^42^ (Fig. 6, Supplementary Figs. 3–10 and Supplementary Table 6). We did not find that trabeculation (mean global fractal dimension) as an exposure had a causal association with cardiomyopathies or heart failure outcomes that was independent of LV remodeling (see Supplementary Table 6 for multivariable MR analyses). However, we identified opposing directions of effects for many trabeculation-associated variants when applied to HCM and DCM, suggesting they modify the phenotype differently in each disease (Fig. 6 and Extended Data Fig. 9). We identified evidence supporting causal effects of genetic liability to DCM with increased trabecular complexity and heart failure. Conversely, genetic liability to HCM was negatively associated with increased trabecular complexity, DCM and heart failure (Fig. 6 and Extended Data Figs. 9 and 10). These findings suggest that shared biological processes contribute to trabeculation and heart failure in both cardiomyopathies but through opposing mechanisms that are remodeling dependent.

## Discussion

Myocardial trabeculae are highly conserved structures in vertebrates where their complex forms are driven by multiscale interactions during development^43^. Disrupting their development in animal models is frequently lethal, but their role in adults is not fully understood and may relate to maintaining efficient intraventricular flow patterns and force transmission in health and disease^5,17^. Here, we analyze myocardial trabecular traits in adult humans across the allele frequency spectrum discovering loci regulating chamber maturation, complex biological patterning and conduction traits. We found that the same trabeculation-associated common variants had opposing effects in cardiomyopathies, suggesting that they cause distinct phenotypes in different diseases.

Several discovered loci are known to regulate trabecular development and cell fate determination. Common variants mapped to genes in *NRG1*, *NOTCH1* and *NKX2-5*, which regulate trabecular morphogenesis and define the building plan for trabeculation through neuregulin 1*β*/ErbB signaling and modifying cardiac jelly dynamics in the developing heart^2^. The gene of the zinc finger transcription factor *GATA6* is expressed early in the developing heart and is critical for activation of the cardiomyocyte gene expression program^44^. Of note, the *MIB1* gene is located upstream of *GATA6* and encodes an E3 ubiquitin ligase that promotes endocytosis of NOTCH ligands. Variants in this gene cause hypertrabeculation in an autosomal-dominant fashion and inactivation causes hypertrabeculation in mice^45^. The Hand proteins are also essential regulators of cardiac development, and HAND2 is a crucial downstream transcriptional effector of endocardial Notch signaling during embryonic trabeculation^46^. The replicated common variant loci in *GOSR2* and *MTSS1* regulate cytoskeletal branching and may increase structural surface area in different organ systems^17,47,48^. Newly discovered loci in *CEP85L* and *PKD1* suggest there may also be a role for cilia and microtubule organization in trabecular formation—potentially through endocardial mechanosensing and signaling^49,50^.

Trabeculation is a dynamic phenotype that responds to transient changes in loading conditions^6,7^, but the pathophysiological interactions between trabeculation and remodeling in cardiomyopathy are not known. We found that increased trabeculation can be evident at presentation in some patients with cardiomyopathy, suggesting that additional common or rare genetic modifiers may contribute to the phenotype^3^. We identified individually rare PAVs, including nonsarcomeric genes, that could be plausible candidates for such modifiers. We did not find that aggregating common variants, as an instrument for trabeculation, led to an association with outcomes when pruning cardiomyopathy-associated variants. However, this relationship may be better understood by the finding that many trabeculation-associated variants show opposing directions of effects depending on the underlying cardiomyopathy, suggesting they modify the phenotype differently in each disease. Variants in the gene *CDKN1A*, a nonsarcomeric gene that regulates stress-induced remodeling^51^ and a regulator of cell-cycle progression, used as an instrument for trabeculation showed the strongest divergent effects on HCM and DCM as outcomes. There are genetic correlations between other LV traits and cardiomyopathies that also have opposing effects in HCM and DCM^52^. Together, these findings suggest that changes in trabeculation may occur early in the pathogenesis of heart disease and can be modified by genes regulating pathways outside the sarcomere, and causality depends on the underlying cardiomyopathic substrate.

Increased trabeculation is a characteristic of both HCM and DCM, but for some genes, the phenotypic associations may be predominantly associated with isolated hypertrabeculation phenotypes^3^. We identified several genes associated with trabeculation that also have evidence for what is termed as left ventricular noncompaction cardiomyopathy (*MYH7*, *MIB1* (*GATA6*), *DES*, *MYBPC3*, *TTN*, *ACTN2*, *LMNA*, *PLN*, *TBX20*, *TBX5*, *DTNA* and *TNNT2*)^3,53,54^. The descriptive label for the phenotype is based on the proposed mechanism of arrested incorporation of trabeculae into the compacted wall during mammalian heart development^55^, although there is no direct evidence for such compaction in the developing human heart^56^. It has been previously reported that asymptomatic individuals with hypertrabeculation have a normal life expectancy, while those who are symptomatic with hypertrabeculation are at risk of adverse cardiac events and heart failure^10^. We found that both the lowest and highest extremes of trabeculation were associated with a greater risk of heart failure outcomes. Increased trabeculation was associated with a greater risk of future structural and conduction disease even in hearts that were not remodeled at baseline. The strongest risk factors for increased trabeculation were African ancestry, wearable-measured physical activity, harboring cardiomyopathy-associated pathogenic/likely pathogenic (P/LP) variants or a diagnosis of overt disease.

A limitation of the current analysis is that it is at a single time point for each participant, so we do not know the temporal trajectory of trabecular adaptation and the relationship with remodeling. UK Biobank is a large-cross sectional study that is subject to selection bias and latent population stratification; however, risk factor associations appear to be broadly generalizable^57^. The population is predominantly European, and further work is needed to understand variation in trabecular morphology in people of diverse ancestries. We could also not fully account for differential cohort and periodic effects. Causal inferences were limited to the MR studies and reflect the limitations of population-based observational data. Image phenotyping combined deep learning myocardial segmentation with classical edge detection to quantify trabecular complexity; however, the distribution of values will have a dependency on the parameters and processing steps performed.

In conclusion, genetic variants across the allele frequency spectrum are associated with trabecular traits in the mature human heart pointing to pathways that orchestrate early organ development, determine cell-fate commitment and regulate cytoskeletal complexity. Trabeculation-associated loci may also act as risk factors or disease modifiers in cardiomyopathy but have different effects depending on the genetic substrate. Myocardial trabeculae are an intricate and dynamic phenotype, and these findings reveal mechanisms of how these complex tissue forms emerge and their importance to adult health and disease.

## Methods

### Study overview

The UK Biobank study recruited 500,000 participants aged 40–69 years old from across the United Kingdom between 2006 and 2010 (National Research Ethics Service, 11/NW/0382)^22^. Genotyping array data and exome sequencing data were available for over 450,000 participants. This study was conducted under the terms of access of projects 40616 and 47602. All participants provided written informed consent. A substudy recalled participants for CMR imaging^23^, and volumetric traits were measured using quality-controlled deep learning algorithms^24–26^. Imaging was made available in two releases that formed a discovery group of 38,245 and a validation group of 9,558 participants.

### Fractal analysis of trabecular morphology

Deep neural networks were used for short-axis cine end-diastolic left ventricular segmentation via a fully convolutional network to label pixels containing myocardium. The performance of image annotation using this algorithm is equivalent to a consensus of expert human readers and achieves subpixel accuracy for cardiac segmentation^24^. For each slice, a region of interest was defined within the midwall of the LV myocardium between the automated endocardial and epicardial segmentations. Subsequent image processing consisted of bias-field correction using histogram stretching, applying a region-based level-set algorithm to segment the trabeculations and create a binary mask^58^. The mask was then converted to an outline using a Sobel filter, and fractal dimension was calculated using a standard box-counting method in which the target image is overlaid by a grid of known box size and the number of boxes containing nonzero image pixels is recorded (Supplementary Fig. 13). This process is repeated with box sizes between two pixels and 45% of the image size. Fractal dimension is defined as the negative gradient of an ordinary least-squares fit line to the logarithm of box size and box count. Fractal analysis was automated using FracAnalyse software^16,18^ and adapted for the scale of the UK Biobank and derived segmentations (AutoFD^17^). A fractal dimension is a scale-invariant ratio providing an index of complexity. After analysis of AutoFD and FracAnalyse on the segmented UK Biobank images^17,18^, the data were interpolated. The raw fractal dimension data were interpolated using a Gaussian kernel local fit to a nine-slice template to allow for comparison across subjects (fracDecimateFD.R; interpNoSlices = 9, with a minimal slice cutoff of 6 (cut.off = 6)). Summary statistics (minimum, mean, median and maximum) were calculated from the nine slices for four cardiac levels: global (1–9 slices), basal (1–3 slices), mid (4–6 slices) and apical (7–9 slices) using summaryFD.R (sections = BMA, discard = FALSE). The minima, maxima and medians were calculated using rowMins, rowMaxs and rowMedians, respectively, in R. The analysis was performed on each left ventricular slice. We did not differentiate between trabeculae and papillary muscles as edge detection was applied within the whole LV cavity.

The measures were adjusted for covariates: age at scan, age^2^, reported sex, age:sex interaction, imaging center, body surface area, automatic SBP, days per week of vigorous exercise, and ten genetic principal components for ancestry, through multiple linear regression producing standardized residuals (mean 0, s.d. 1; Supplementary Table 1). Self-reported ancestry was used to assess any difference in trabecular morphology by ancestry.

### ECG diagnoses

The ECGs were performed according to a defined protocol and analyzed using proprietary software (GE CardioSoft). Data from the first imaging visit (instance 2, *n* = 42,386) were labeled using a previously trained convolutional neural network^29^ designed to identify six diagnoses from the ECG: sinus bradycardia, sinus tachycardia, left bundle branch block, right bundle branch block, first-degree AV block and atrial fibrillation. Automated diagnoses had F1 scores above 80% and specificity over 99% (ref. ^29^). The ECGs were preprocessed with a bandpass filter 0.5 to 100 Hz, a notch filter at 60 Hz and resampling to 400 Hz. Zero padding resulted in a signal with 4,096 samples for each lead for a 10 s recording, which was used as input to the neural network model. The binary outputs (presence or absence of each diagnosis) were used for subsequent analyses.

### Rare and common genetic association studies

Genotype calling was performed on the UK BiLEVE Axiom array and the UK Biobank Axiom array, resulting in 805,426 markers in GRCh37 coordinates, and quality control was performed^22^. The dataset was phased, and 96M genotypes were imputed using the Haplotype Reference Consortium and UK10K haplotype resources^22^. Exome sequencing was performed on the Illumina NovaSeq 6000 platform. Reads were mapped to the hg38 reference genome, and quality control was performed^59^.

RVASs were carried out using Regenie software on the DNA Nexus Research Analysis Platform. The raw genotyping data for step 1 of Regenie were lifted from GrCh37 to GrCh38 using picard and bcftools. SNPs in autosomes with a minor allele frequency <0.01, missingness >0.01, a minor allele count <20 or deviations from the Hardy–Weinberg equilibrium (5 × 10^−15^), and individuals with more than 10% missingness, were excluded from the analysis. Interchromosome, SNPs in linkage disequilibrium (indep-pairwise 1000 100 0.9) and areas of low complexity were excluded for step 1. Exome sequencing data for step 2 were quality controlled for variants in the autosomes with missingness less than 10%, variants where less than 90% of all genotypes for that variant had a read depth less than 10, deviations from the Hardy–Weinberg equilibrium (1 × 10^−15^) and individuals with more than 10% missingness. Step 2 of Regenie was run for the 25 trabeculation summary measures over different allele frequencies (singletons, 0.01, 0.001) for 6 overlapping PAV custom masks (predicted loss-of-function variants (LoF) only; missense only (flagged by >1 of 5 deleterious software); missense only (all); missense only (flagged by all of 5 deleterious software); PAVs (LoF and missense flagged by >1 of 5 deleterious software); and PAVs (all LoF and missense)), where the minimum minor allele count was at least 3. Bonferroni significance for 18,117 included genes was *P* < 2.76 × 10^−6^, and the genes of the statistically significant RVAS results in 29,480 participants were validated (*P* < 0.05) in a holdout group of 7,293 participants. A full analysis was also completed by combining the discovery and validation cohorts.

For GWAS, the imputed UK Biobank genotyping data were converted from bgen format to plink using plink2. Minor allele frequency of >0.001 in autosomes was included. Individuals with more than 5% missing genotypes and SNPs with more than 5% missingness were excluded. Participant sex discrepancies, heterozygosity and relatedness were handled by keeping only European individuals and participants included in the UK Biobank principal components analysis^17,22^. SNPs deviating from the Hardy–Weinberg equilibrium (1 × 10^−8^) and those with an imputation quality score (INFO) of <0.4 were excluded. Individuals with trabeculation data were extracted, and GWAS was undertaken for 30,419 participants using Genome-wide Complex Trait Analysis (GCTA) software (version 64). A sparse genetic relationship matrix (GRM) was created and FastGWA was undertaken with a mixed linear model, adjusting for the genotyping array batch (and discovery or validation batch in the full analysis). Twenty-five trabeculation summary traits were analyzed, and the statistically significant GWAS results (*P* < 5 × 10^−8^) were validated (*P* < 0.05) in a holdout group of 7,593 participants. A full analysis was also completed by combining the discovery and validation cohorts (Supplementary Tables 5 and 9). Genes of independent loci were prioritized through LocusZoom, expression quantitative trait loci from GTEx v8 and TWAS. TWAS with S-MultiXcan^60^ was completed using the GWAS summary statistics from GTEx V8 that identified 128 cardiac-specific (LV and atrial appendage) genes and 336 genes from all tissues that were significantly associated with trabeculation after correction for the number of tested genes (Supplementary Tables 11 and 12)^30,31^. Phenotype associations through GWAS catalog, Phenoscanner and PheWEB were assessed (Supplementary Tables 5 and 9), and resulting genes were analyzed by GO resource enrichment analysis using Panther (Supplementary Table 13).

### Cardiomyopathy-associated variant curation

Cardiomyopathy-associated rare variants were identified^25,26^ for HCM and DCM. Individuals were classified as genotype negative (SARC-NEG) if they had no rare protein-altering genetic variation (minor allele frequency <0.001 in the UK Biobank and the Genome Aggregation Database (gnomAD v2.1)) in any genes that may cause or mimic HCM or DCM. These genes represented an inclusive list of genes with definitive or strong evidence of an association with CM, moderate evidence and genes associated with syndromic phenotypes^38,61,62^. This SARC-NEG group was compared with individuals with disease-associated rare variants in genes with strong or definitive evidence for HCM (*MYBPC3*, *MYH7*, *MYL2*, *MYL3*, *TNNI3*, *TNNT2*, *TPM1* and *ACTC1*) and DCM (*BAG3*, *DES*, *DSP*, *FLNC*, *LMNA*, *MYH7*, *PLN*, *RBM20*, *SCN5A*, *TNNC1*, *TNNT2* and *TTN*). Analysis was restricted to robustly disease-associated variant classes for each gene and to variants sufficiently rare to cause penetrant disease (filtering allele frequency <0.00004 for HCM and 0.000084 for DCM^63^). Variants were classified as P/LP (SARC-P/LP) if reported as P/LP for cardiomyopathy in ClinVar and confirmed by manual review.

For HCM, variants in the region of 29 HCM or left ventricular hypertrophy-associated genes were extracted from the whole-exome sequencing data of 454,756 participants. Matched Annotation from NCBI and EMBL-EBI transcripts, PAVs that had a minor allele frequency <0.1% in gnomAD and UK Biobank, were identified. The UK Biobank exome data were annotated using Ensembl Variant Effect Predictor (version 105) with a plugin for gnomAD (version r2.1), Loss-Of-Function Transcript Effect Estimator (LOFTEE) and SpliceAI. Intron variants that were pathogenic in ClinVar were manually curated for functional evidence of splicing. Splice region variants and other splice variants (excluding essential splice and splice donor fifth base) were included if they were predicted to cause splicing by SpliceAI (threshold >0.8). The variant list was then shortened to include only the eight definitive evidence HCM genes (*MYBPC3*, *MYH7*, *TNNT2*, *TNNI3*, *TPM1*, *MYL2*, *MYL3* and *ACTC1*). LOFTEE was used to identify low-confidence LoF variants and identify PAVs that are mislabeled (for example, NAGNAG sites). The variants were then filtered for disease-causing mechanisms. All MYBPC3 PAVs were kept; for the other seven genes, only variants influencing gene product structure (indels, missense, start and stop lost) or gene product level but nonsense-mediated mRNA decay escaping were kept. The variant list was then shortened to include only variants that met a filtering allele frequencing of <0.00004 in gnomAD popmax faf and as a minor allele frequency in UK Biobank. The variant list was then shortened to include variants that would be called P/LP if identified in a patient with HCM; using ClinVar, the variants were manually curated if they had any evidence of pathogenicity.

For DCM, extraction was similar to the HCM variant curation pipeline but for 19 DCM-associated genes with definitive, strong or moderate evidence for disease. Only truncating variants in *TTN* in percentage spliced in >90% exons and variants with a splice prediction or curation of pathogenic (with assessment) were included. The variant list was then shortened to include only the 12 definitive or strong evidence DCM genes (*BAG3*, *DES*, *DSP*, *FLNC*, *LMNA*, *MYH7*, *PLN*, *RBM20*, *SCN5A*, *TNNC1*, *TNNT2* and *TTN*). The variants were filtered for disease-causing mechanisms. All variants in *BAG3*, *LMNA*, *PLN*, *SCN5A*, *RBM20* and *DSP* PAVs were kept; only variants influencing gene product structure (indels, missense, start and stop lost) or gene product level but nonsense-mediated mRNA decay escaping were kept for *MYH7*, *DES*, *TNNC1* and *TNNT2*; only variants predicted to influencing gene product level (for example, frameshift, splice and stop gained) were kept for *TTN* and *FLNC*. In addition, compound heterozygous carriers of common truncating variants in *TTN* (same two TTN variants identified in more than ten individuals) were removed from the analysis as these are probably rescued.

### Phenotypes and clinical features

Metabolic equivalents (MET) data were summarized in three variables: summed MET minutes per week for all activity, above moderate/vigorous recommendation, and MET minutes per week for vigorous activity. For PheWASs, the 2021 data release of refreshed HES was used to identify the ICD codes and patients of the conditions. PheWAS were undertaken using the PheWAS R package with clinical outcomes and coded phenotypes converted to 1,840 categorical PheCodes. PheWAS was undertaken with the full cohort of 47,803 participants, and all traits were adjusted using multiple linear regression for age at scan, age^2^, sex, age:sex interaction, imaging center, body surface area, SBP, vigorous exercise via questionnaire (days per week), and 10 genetic principal components of ancestry. *P* values were deemed statistically significant with Bonferroni adjustment for the number of PheCodes. The UK Biobank provided the first occurrence of health outcomes defined by a three-character ICD10 code using hospital in-patient, death and primary care records, which were used for cumulative hazard curves. Cumulative hazard curves were undertaken with the full cohort of 47,803 participants for mean global trabeculation and created using the first reported UK Biobank data (summarizing the first date of report from all UK Biobank data (HES, primary care, self-reported and so on)) and the survival and survminer R packages from date of imaging to death or diagnosis date. Participants diagnosed with cardiomyopathy or heart failure before imaging were excluded. Hyper- and hypotrabeculation were deemed >1.5 or <1.5 s.d. from the mean with sensitivity analyses also completed for 1 s.d., 2 s.d. and deciles.

### Additional genetic analyses

MR was assessed using GWAS summary statistics from published literature^30–32^ with mean global fractal dimension using the R packages TwoSampleMR and MVMR. Exposure variants were included if GWAS significant (*P* < 5 × 10^−8^). The GWAS-significant loci of mean global fractal dimension in the full cohort (*n* = 38,012 European participants) were used to test causality for trabecular morphology in DCM, HCM and heart failure, with and without adjustment for LVEDV. Tests of pleiotropy, Steiger directionality and heterogeneity were assessed (Supplementary Figs. 3–10 and Supplementary Table 6). Heritability was estimated by creating a GRM in GCTA and using a restricted maximum likelihood analysis to estimate the variance explained by the SNPs that were used to estimate the GRM (Supplementary Table 3). Genetic correlations were assessed using –reml-bivar in GCTA for two traits (Supplementary Table 2). PRS for HCM and DCM was created previously from GWAS summary statistics and applied to the UK Biobank cohort^30,32,64^. SNPs from the GWAS of mean global fractal dimension of 30,000 European participants were assembled into a PRS^65^ and tested on 10,000 European participants with fractal dimension available for comparison. The best threshold was 0.000001, where *R*^2^ = 0.02 and *R* = 0.15, similar to other cardiomyopathy-associated PGS (for example, HCM (*R*^2^ = 0.03–0.05))^64^. The PRS did not have a statistically significant association with a diagnosis of cardiomyopathy. Categorical variables were assessed using the chi-squared test or Fisher’s exact test. Continuous variables were assessed using two-sided Student’s *t*-test.

### Reporting summary

Further information on research design is available in the Nature Portfolio Reporting Summary linked to this article.

## Supplementary information

Supplementary Information Supplementary Figs. 1–13.

Reporting Summary

Supplementary Table 1–13: Supplementary Table 1: Model information from the multiple linear regression of covariates when adjusting mean global trabeculation. For multiple comparisons, *P* < 0.001 was deemed statistically significant. *t*, two-sided *t*-test. Supplementary Table 2: Genetic covariance (covg) and correlation (rg) with mean global trabeculation. Mean global trabeculation had a heritability estimate of 43%. The estimates are shown with and without adjustment for covariates. The analyses were completed on 38,245 participants of the UK Biobank population. Supplementary Table 3: Summary information on the two groups of imaged UK Biobank participants and heritability estimates for trabeculation measures. Slice, imaging slice; Min, minimum; Med, median; FD, fractal dimension measure of trabeculation. Supplementary Table 4: Inflation factor from GWAS. Slice, image slices. Supplementary Table 5: The table depicts the independent statistically significant GWAS loci for trabecular measures as observed from LocusZoom. name, random ID of independent locus; nocmhf?, whether the locus was also observed when participants in the UK Biobank with cardiomyopathy or heart failure were removed; nocm?, whether the locus was also observed when participants in the UK Biobank with cardiomyopathy were removed; Region, position of locus in GrCh38; Gene1-3; nearest genes from LocusZoom. Supplementary Table 6: Summary of MR results. The table depicts the results for trabeculation as outcome and exposure with DCM, HCM and heart failure (HF). Five statistical methods were used to assess for causality, with the number of SNPs included in the analysis (nsnp), beta effect size (b), s.e.m. and *P* value (pval) shown. For multiple comparisons, *P* < 0.001 was deemed statistically significant. Supplementary Table 7: The table depicts the validated RVAS results for trabecular measures. CHROM, chromosome; GENPOS, gene position; ALLELE1, masks; A1FREQ, allele frequency; *N*, sample size of discovery cohort; TEST, RVAS method analyzed by Regenie software; LOG10P, *P* value of association. Supplementary Table 8: The table depicts the PheWAS results for linear regression with imaging and trabecular measures. For multiple comparisons, Bonferroni correction for the number of phenotypes analyzed was applied. OR, odds ratio; *P*, *P* value of association; n_total phenotype, number of participants with imaging measure. Supplementary Table 9: The table depicts the novel, independent, statistically significant GWAS loci for trabecular measures for both validation and discovery datasets as observed from LocusZoom. name, random ID of independent locus; Region, position of locus in GrCh38; Gene1-3; nearest genes from LocusZoom. Supplementary Table 10: The table depicts the novel, RVAS results for trabecular measures using both discovery and validation cohorts. CHROM, chromosome; GENPOS, gene position; ALLELE1, masks; A1FREQ, allele frequency; *N*, sample size of discovery cohort; TEST, RVAS method analyzed by Regenie software; LOG10P, *P* value of association. Supplementary Table 11: Statistically significant TWAS multivariate regression results for trabecular measures using both discovery and validation cohorts for cardiac tissues only (LV and atrial appendage). For multiple comparisons, a Bonferroni correction for the number of genes analyzed was applied. Supplementary Table 12: Statistically significant TWAS multivariate regression results for trabecular measures using both discovery and validation cohorts. For multiple comparisons, a Bonferroni correction for the number of genes analyzed was applied. Supplementary Table 13: Statistically significant GO resource enrichment analysis results using Panther on all genes in Supplementary Tables 5, 7, 9 and 10. A PANTHER overrepresentation test (released 17 October 2023) was performed on the GO Ontology human database (https://geneontology.org/, released 17 January 2024). Statistical significance was assessed via Fisher exact test with a false discovery rate correction for multiple comparisons. The GO gene lists assessed are shown in bold.

## Data Availability

The derived phenotypes from this analysis are available from UK Biobank (https://biobank.ndph.ox.ac.uk/showcase/; upload identifier 5777). Summary statistics are available from the GWAS catalog (https://www.ebi.ac.uk/gwas/) accession numbers GCST90449116 to GCST90449140. GWAS-significant SNPs are accessible on LocusZoom (https://my.locuszoom.org/gwas/634035/).

## Code availability

The analysis code is available on GitHub at https://github.com/ImperialCollegeLondon/trabecular_variants.
