# Supplementary material for: Genetic and phenotypic architecture of human myocardial trabeculation
Source: Nat Cardiovasc Res. 2024 Nov 20;3(12):1503–15. doi: 10.1038/s44161-024-00564-3 (PMC11634767; doi:10.1038/s44161-024-00564-3)
Supplement: Supplementary file 1 — Supplementary Figs. 1–13. [file 44161_2024_564_MOESM1_ESM.pdf]

nature cardiovascular research

Supplementary information https://doi.org/10.1038/s44161-024-00564-3

Genetic and phenotypic architecture of human myocardial trabeculation

In the format provided by the authors and unedited

Supplementary information

List of Figures

1 **Association of alcohol intake and average acceleration with mean global trabeculation.** The presented box plots visualise the median, first and third quartiles and the mean is presented as a diamond. Student's two-sided t-test was used to compare the group means. The stars represent the p-value. ns, not significant, p>0.05; *, p<=0.05; **, p<=0.01; ***, p<=0.001; ****, p<=0.0001. For multiple comparisons, a p<=0.001 was deemed significant. Covariate-adjusted mean global trabeculation measured by fractal dimension analysis was compared to **A)** alcohol intake and the middle group (10%-90%; mid, n=23,201) compared to the lowest 10% (low10%, n=2,847) of alcohol drinkers showed significantly decreased trabecular complexity in the lowest group and significantly increased complexity in the top drinkers (top10%, n=2,892). The comparison with **B)** showed that trabeculation had a positive relationship with acceleration measures via accelerometer; comparing the middle group (10%-90%; mid, n=13,701) to the lowest 10% (low10%, n=1,711) and the top 10% (top10%, n=1,713). Summed metabolic equivalent (MET) minutes per week for all activity (data ID 22040.0.0, highest compared to lowest 10%, R=0.03; β=0.11, SE=0.01, P=2.2e-05), for vigorous activity (data ID 22039.0.0, R=0.04; β=0.13, SE=0.01, P=1.9e-09), and above moderate/vigorous recommendation (data ID 22035.0.0, β=0.08, SE=0.01, P=7.6e-12), as well as reported exercise via questionnaire (days per week (0 days versus 7 days); moderate exercise β=0.11, SE=0.02, P=6.7e-09; vigorous exercise β=0.19, SE=0.03, P=1.3e-07) and overall acceleration average via accelerometer data (R=0.06; β=0.24, SE=0.01, P=1.2e-12), associated with increased trabecular complexity, with the effect more substantial for activity deemed vigorous. The association with physical activity was in part due to LVEDV: adjustment for LVEDV retained significance only for vigorous exercise (reported via questionnaire as days per week (0 days versus 7 days), β=0.11, SE=0.05, P=0.03). 3

2 **Adjustment for LVEDV makes little difference to the meta Manhattan plot of the minimum p-value for all SNPs across all TM measures.** The Manhattan plots depict the p-value of association (x-axis, -log10(p-value) and the SNPs across 22 chromosomes (y-axis) from a GWAS of 38,245 participants of the UK Biobank population. The GWAS results were significant if P<5x10-8. 3

3 **Mendelian randomization analysis of HF as exposure on mean global FD.** The plots show summary information on the analyses, performed as per the TwoSampleMR R package. FD SNPs were included from the GWAS results of 38,245 European participants and compared to publicly available GWAS results (see Methods). Error bars represent standard error. 4

4 **Mendelian randomization analysis of DCM as exposure on trabeculation outcome.** The plots show summary information on the analyses, performed as per the TwoSampleMR R package. FD SNPs were included from the GWAS results of 38,245 European participants and compared to publicly available GWAS results (see Methods). Error bars represent standard error. 5

5 **Mendelian randomization analysis of DCM as exposure on trabeculation outcome, pruning for an NFIA variant.** The plots show summary information on the analyses, performed as per the TwoSampleMR R package. FD SNPs were included from the GWAS results of 38,245 European participants and compared to publicly available GWAS results (see Methods). Error bars represent standard error. 6

6 **Mendelian randomization analysis of for HCM as exposure on trabeculation outcome.** The plots show summary information on the analyses, performed as per the TwoSampleMR R package. FD SNPs were included from the GWAS results of 38,245 European participants and compared to publicly available GWAS results (see Methods). Error bars represent standard error. 7

7 **Mendelian randomization analysis of for HCM as exposure on HF outcome.** The plots show summary information on the analyses, performed as per the TwoSampleMR R package. The comparisons were completed using publicly available GWAS results (see Methods). Error bars represent standard error. 8

8 **Mendelian randomization analysis of DCM as exposure on HF outcome.** The plots show summary information on the analyses, performed as per the TwoSampleMR R package. The comparisons were completed using publicly available GWAS results (see Methods). Error bars represent standard error. 9

9 **Mendelian randomization analysis of HCM as exposure on DCM outcome.** The plots show summary information on the analyses, performed as per the TwoSampleMR R package. The comparisons were completed using publicly available GWAS results (see Methods). Error bars represent standard error. 10

10 **Mendelian randomization analysis of DCM as exposure on HCM outcome.** The plots show summary information on the analyses, performed as per the TwoSampleMR R package. The comparisons were completed using publicly available GWAS results (see Methods). Error bars represent standard error. 11
11 **Sensitivity analysis of the association of trabeculation outside 1.5 SD with clinical outcomes.** a-c) by 2 SD (i.e., more extreme) for a) heart failure (hypertrabeculation P=0.00055), b) mitral valve disease, and c) bundle branch block. The mean coxfit linear predictors were plotted for trabeculation by group. Confidence intervals and log-rank p-values are depicted. CI, concordance index. 12
12 **Sensitivity analysis of the association of trabeculation outside 1.5 SD with clinical outcomes.** a-c) by deciles (limited statistical power) for a) heart failure, b) mitral valve disease, and c) bundle branch block. The mean coxfit linear predictors were plotted for trabeculation by group. Confidence intervals and log-rank p-values are depicted. CI, concordance index. 13
13 **Trabecular analysis.** Short axis image example with the level-set outline of the trabeculae, followed by conversion to a binary mask, on which a Sobel filter is applied to generate an outline for box-counting. . . . 13

Supplementary figures

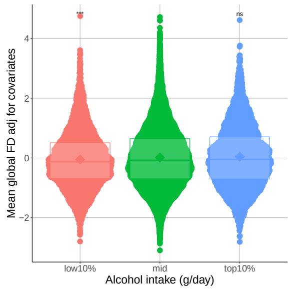

(a) Lowest alcohol drinkers have decreased cardiac trabeculation.

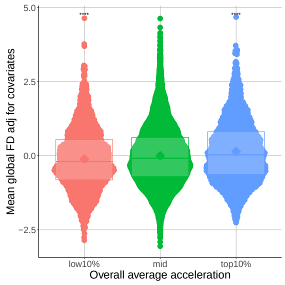

(b) Cardiac trabeculation has a positive relationship with average acceleration.

Supplementary Fig. 1. Association of alcohol intake and average acceleration with mean global trabeculation. The presented box plots visualise the median, first and third quartiles and the mean is presented as a diamond. Student's two-sided t-test was used to compare the group means. The stars represent the p-value. ns, not significant, p>0.05; *, p<=0.05; **, p<=0.01; ***, p<=0.001; ****, p<=0.0001. For multiple comparisons, a p<=0.001 was deemed significant. Covariate-adjusted mean global trabeculation measured by fractal dimension analysis was compared to **A)** alcohol intake and the middle group (10%-90%; mid, n=23,201) compared to the lowest 10% (low10%, n=2,847) of alcohol drinkers showed significantly decreased trabecular complexity in the lowest group and significantly increased complexity in the top drinkers (top10%, n=2,892). The comparison with **B)** showed that trabeculation had a positive relationship with acceleration measures via accelerometer; comparing the middle group (10%-90%; mid, n=13,701) to the lowest 10% (low10%, n=1,711) and the top 10% (top10%, n=1,713). Summed metabolic equivalent (MET) minutes per week for all activity (data ID 22040.0.0, highest compared to lowest 10%, R=0.03; β=0.11, SE=0.01, P=2.2e-05), for vigorous activity (data ID 22039.0.0, R=0.04; β=0.13, SE=0.01, P=1.9e-09), and above moderate/vigorous recommendation (data ID 22035.0.0, β=0.08, SE=0.01, P=7.6e-12), as well as reported exercise via questionnaire (days per week (0 days versus 7 days); moderate exercise β=0.11, SE=0.02, P=6.7e-09; vigorous exercise β=0.19, SE=0.03, P=1.3e-07) and overall acceleration average via accelerometer data (R=0.06; β=0.24, SE=0.01, P=1.2e-12), associated with increased trabecular complexity, with the effect more substantial for activity deemed vigorous. The association with physical activity was in part due to LVEDV: adjustment for LVEDV retained significance only for vigorous exercise (reported via questionnaire as days per week (0 days versus 7 days), β=0.11, SE=0.05, P=0.03).

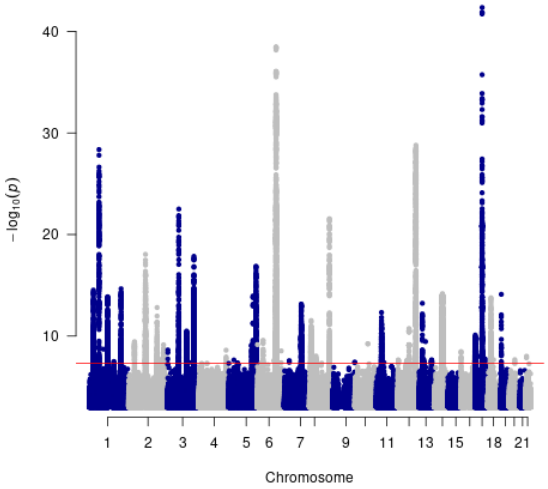

Supplementary Fig. 2. Adjustment for LVEDV makes little difference to the meta Manhattan plot of the minimum p-value for all SNPs across all TM measures. The Manhattan plots depict the p-value of association (x-axis, -log10(p-value) and the SNPs across 22 chromosomes (y-axis) from a GWAS of 38,245 participants of the UK Biobank population. The GWAS results were significant if P<5x10-8.

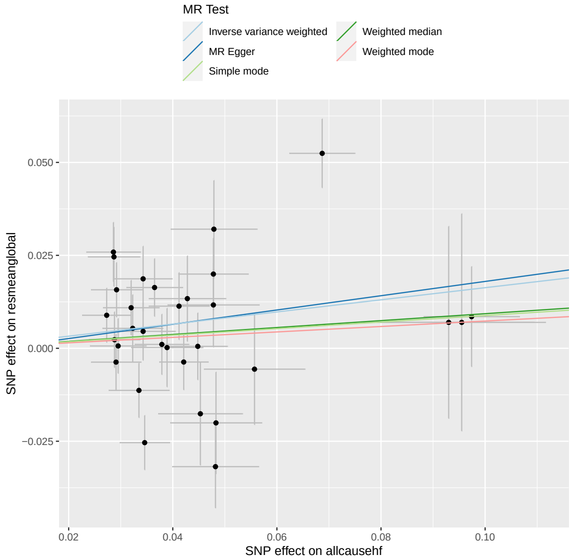

(a) Mendelian randomization scatter plot for HF as exposure on mean global FD.

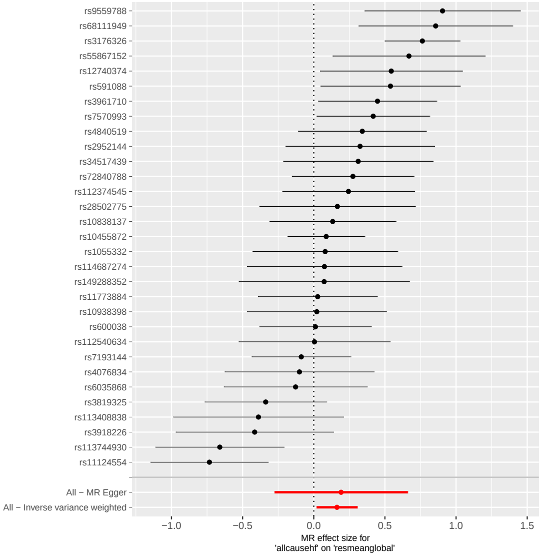

(b) Mendelian randomization single SNP funnel plot for HF as exposure on mean global FD.

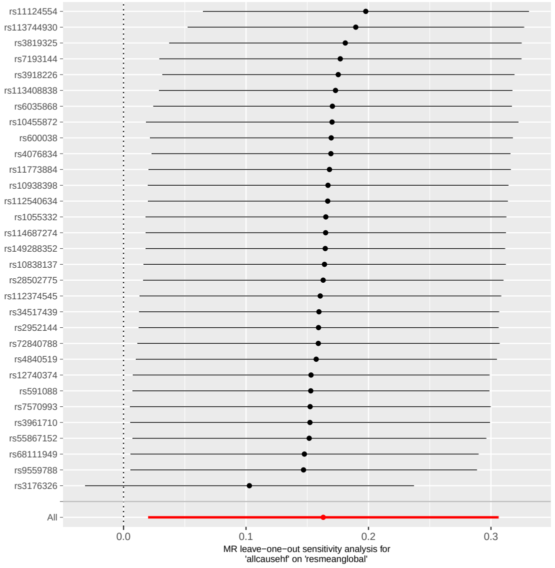

(c) Mendelian randomization single SNP forest plot for HF as exposure on mean global FD.

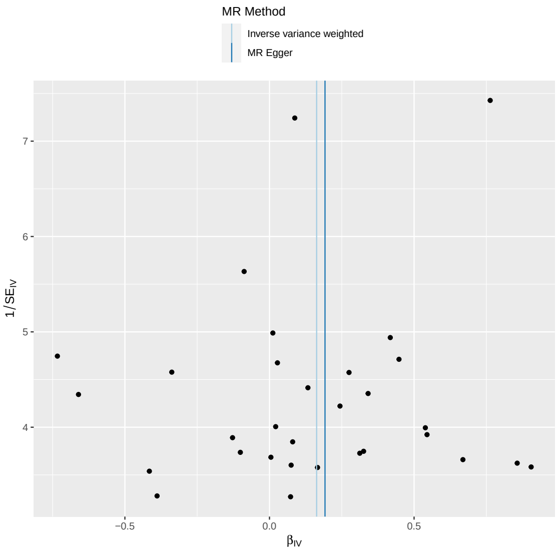

(d) Mendelian randomization leave one out plot for HF as exposure on mean global FD.

Supplementary Fig. 3. Mendelian randomization analysis of HF as exposure on mean global FD. The plots show summary information on the analyses, performed as per the TwoSampleMR R package. FD SNPs were included from the GWAS results of 38,245 European participants and compared to publicly available GWAS results (see Methods). Error bars represent standard error.

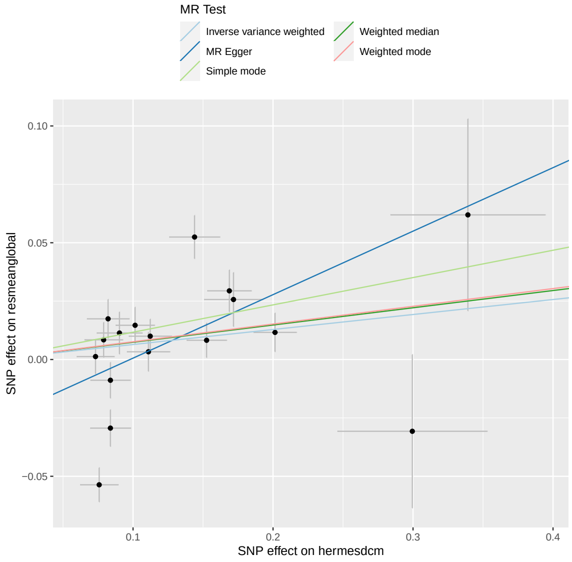

(a) Mendelian randomization scatter plot for DCM as exposure on trabeculation.

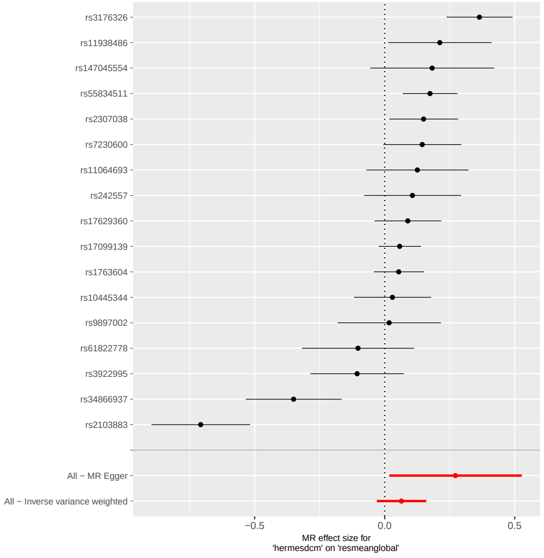

(b) Mendelian randomization single SNP funnel plot for DCM as exposure on trabeculation.

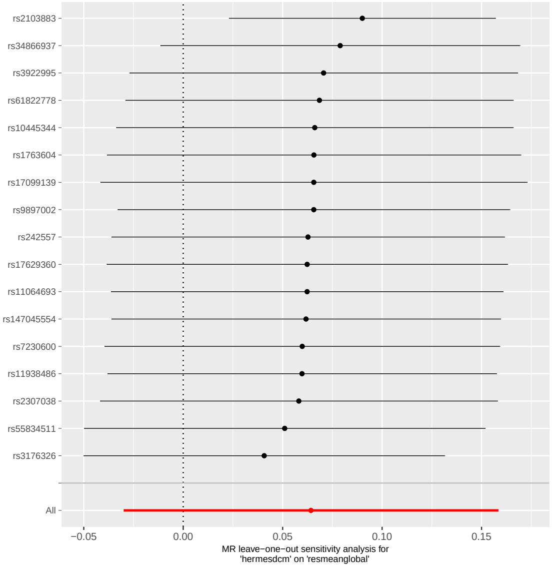

(c) Mendelian randomization single SNP forest plot for DCM as exposure on trabeculation.

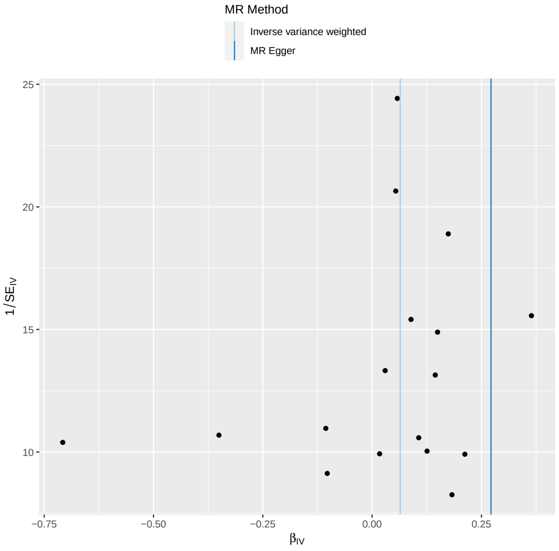

(d) Mendelian randomization leave one out plot for DCM as exposure on trabeculation.

Supplementary Fig. 4. Mendelian randomization analysis of DCM as exposure on trabeculation outcome. The plots show summary information on the analyses, performed as per the TwoSampleMR R package. FD SNPs were included from the GWAS results of 38,245 European participants and compared to publicly available GWAS results (see Methods). Error bars represent standard error.

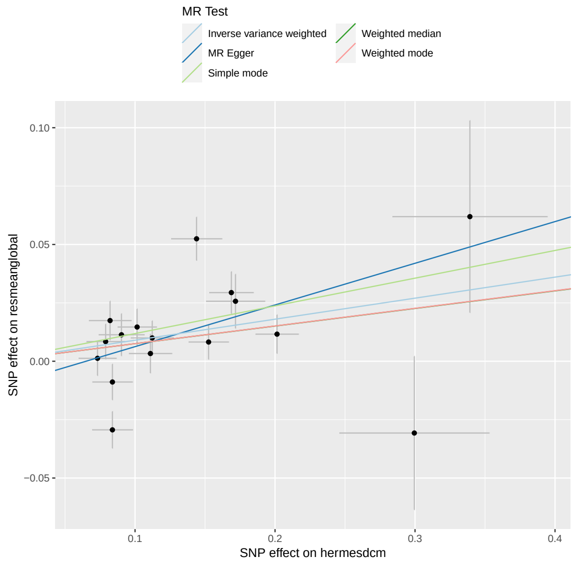

(a) Mendelian randomization scatter plot for DCM as exposure on trabeculation.

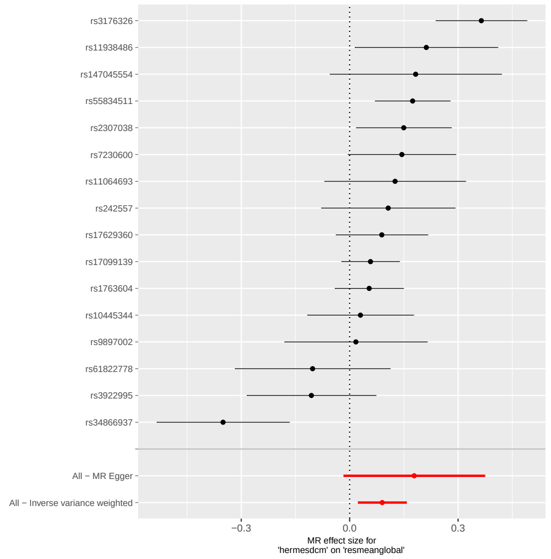

(b) Mendelian randomization single SNP funnel plot for DCM as exposure on trabeculation.

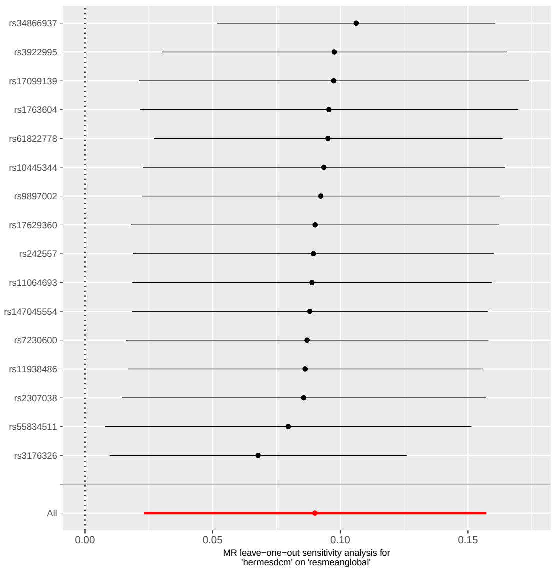

(c) Mendelian randomization single SNP forest plot for DCM as exposure on trabeculation.

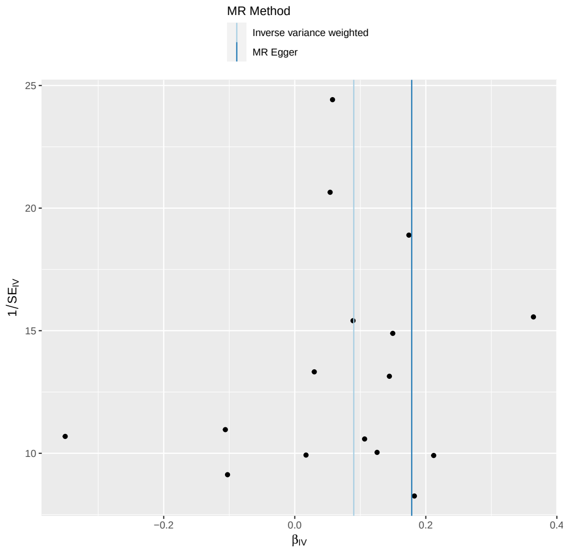

(d) Mendelian randomization leave one out plot for DCM as exposure on trabeculation.

Supplementary Fig. 5. Mendelian randomization analysis of DCM as exposure on trabeculation outcome, pruning for an NFIA variant. The plots show summary information on the analyses, performed as per the TwoSampleMR R package. FD SNPs were included from the GWAS results of 38,245 European participants and compared to publicly available GWAS results (see Methods). Error bars represent standard error.

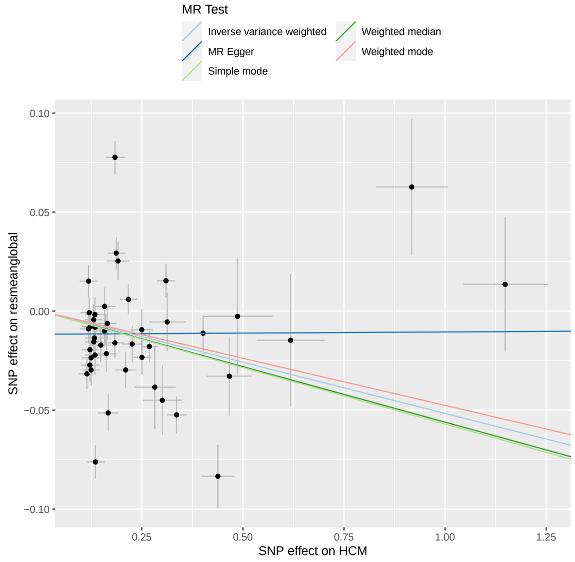

(a) Mendelian randomization scatter plot for HCM as exposure on trabeculation.

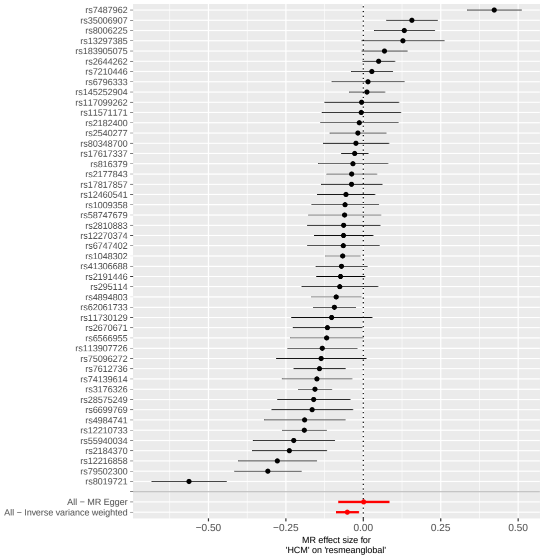

(b) Mendelian randomization single SNP funnel plot for HCM as exposure on trabeculation.

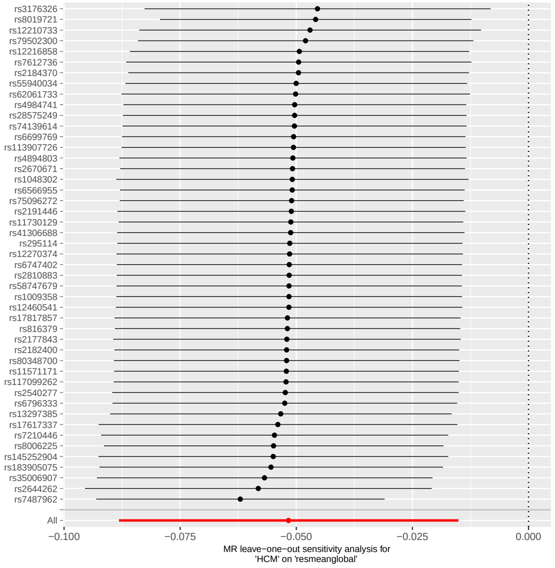

(c) Mendelian randomization single SNP forest plot for HCM as exposure on trabeculation.

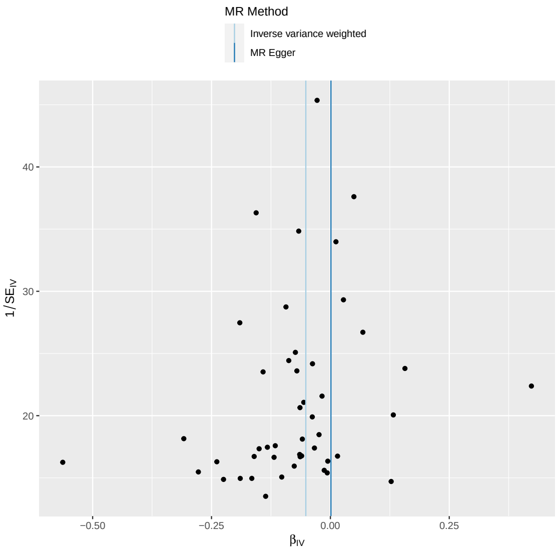

(d) Mendelian randomization leave one out plot for HCM as exposure on trabeculation.

Supplementary Fig. 6. Mendelian randomization analysis of for HCM as exposure on trabeculation outcome. The plots show summary information on the analyses, performed as per the TwoSampleMR R package. FD SNPs were included from the GWAS results of 38,245 European participants and compared to publicly available GWAS results (see Methods). Error bars represent standard error.

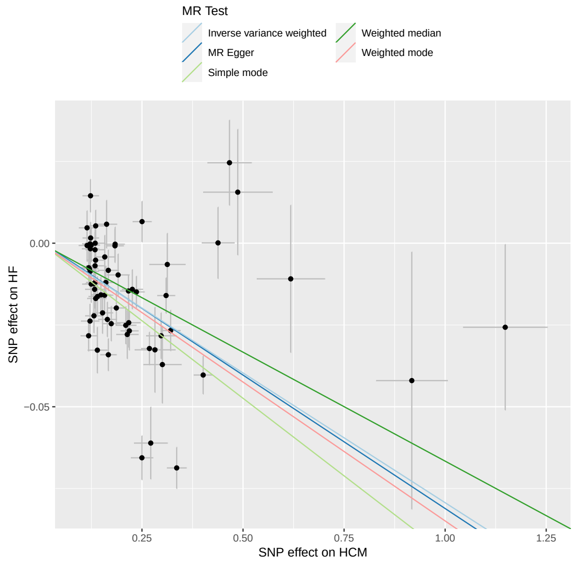

(a) Mendelian randomization scatter plot for HCM as exposure on HF.

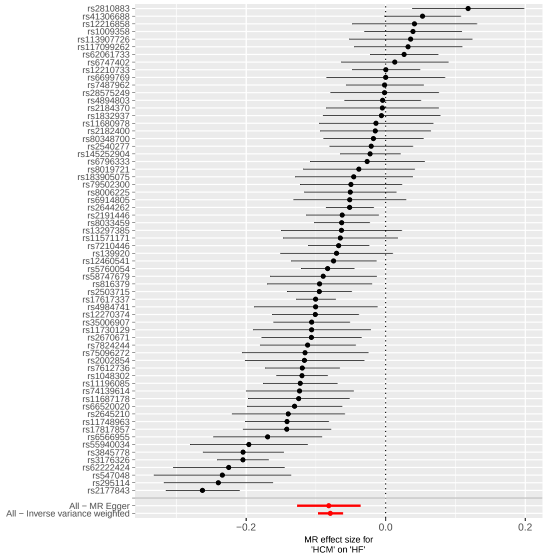

(b) Mendelian randomization single SNP funnel plot for HCM as exposure on HF.

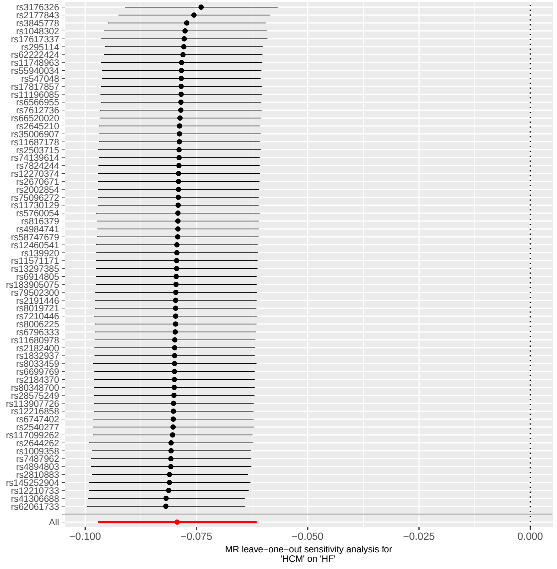

(c) Mendelian randomization single SNP forest plot for HCM as exposure on HF.

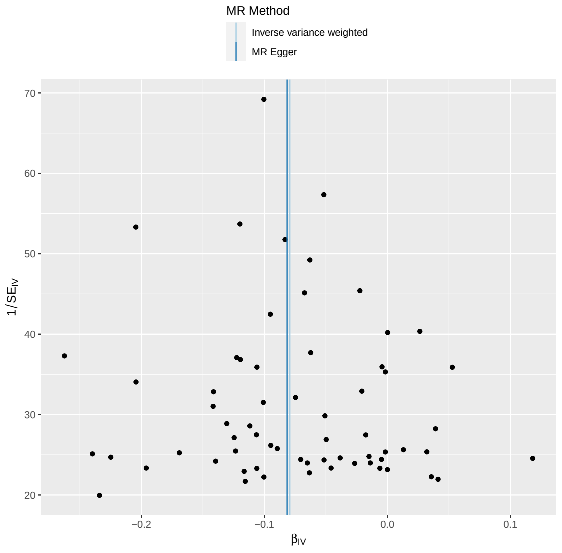

(d) Mendelian randomization leave one out plot for HCM as exposure on HF.

Supplementary Fig. 7. Mendelian randomization analysis of for HCM as exposure on HF outcome. The plots show summary information on the analyses, performed as per the TwoSampleMR R package. The comparisons were completed using publicly available GWAS results (see Methods). Error bars represent standard error.

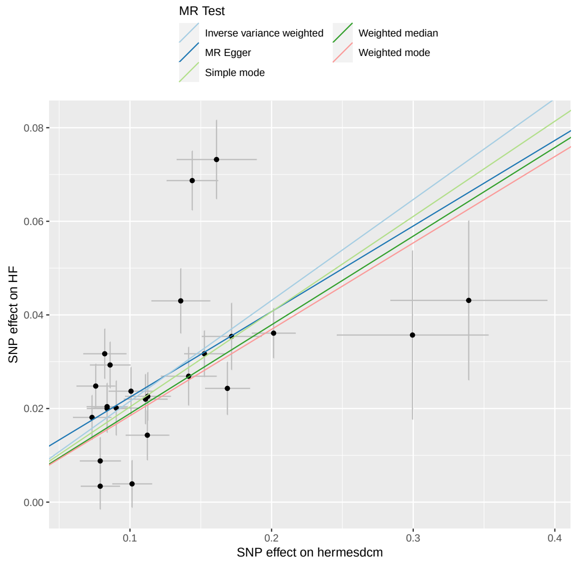

(a) Mendelian randomization scatter plot for DCM as exposure on HF.

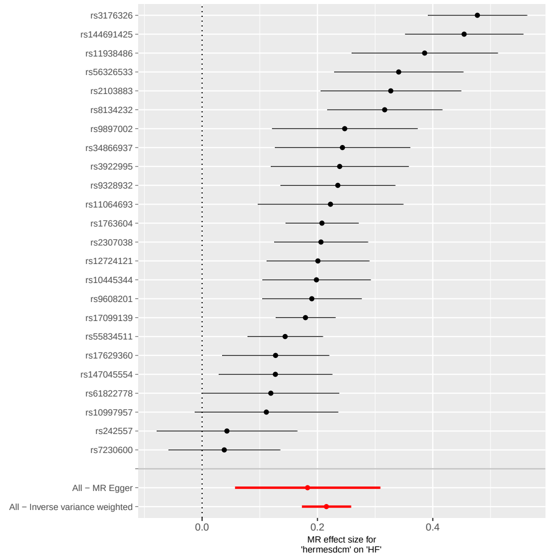

(b) Mendelian randomization single SNP funnel plot for DCM as exposure on HF.

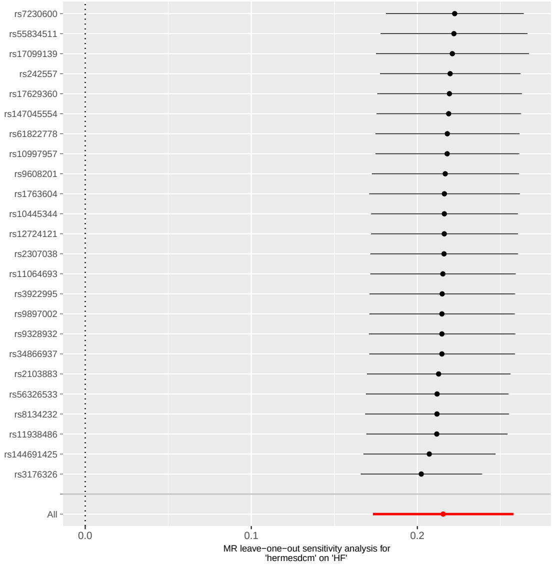

(c) Mendelian randomization single SNP forest plot for DCM as exposure on HF.

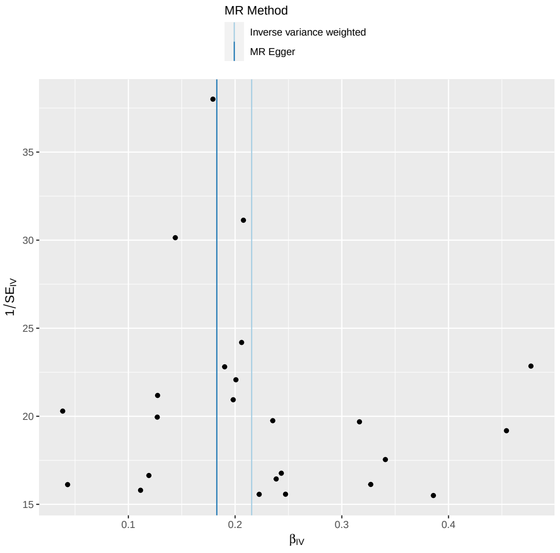

(d) Mendelian randomization leave one out plot for DCM as exposure on HF.

Supplementary Fig. 8. Mendelian randomization analysis of DCM as exposure on HF outcome. The plots show summary information on the analyses, performed as per the TwoSampleMR R package. The comparisons were completed using publicly available GWAS results (see Methods). Error bars represent standard error.

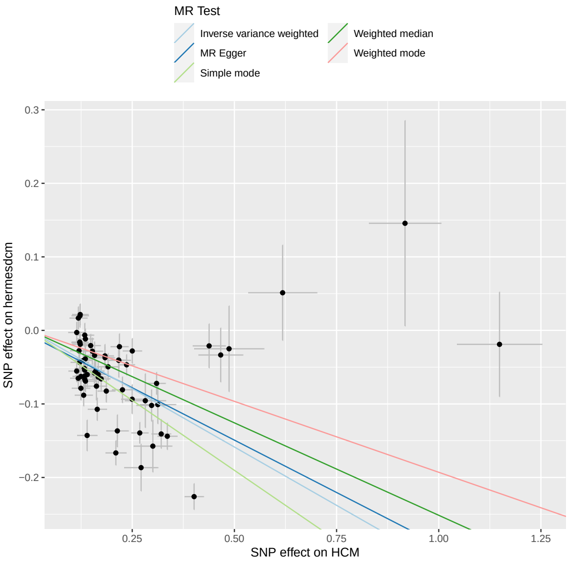

(a) Mendelian randomization scatter plot for HCM as exposure on DCM.

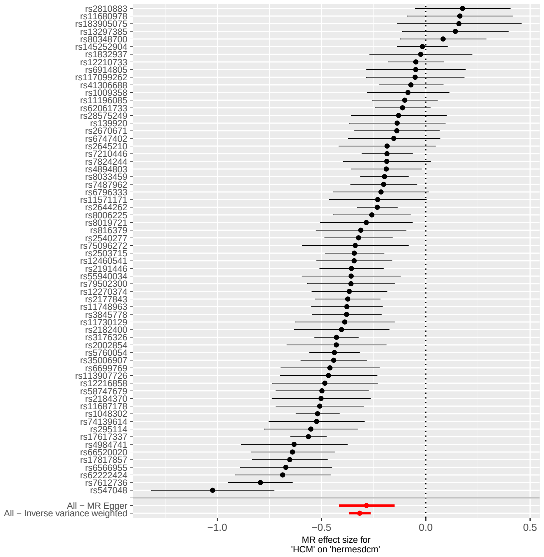

(b) Mendelian randomization single SNP funnel plot for HCM as exposure on DCM.

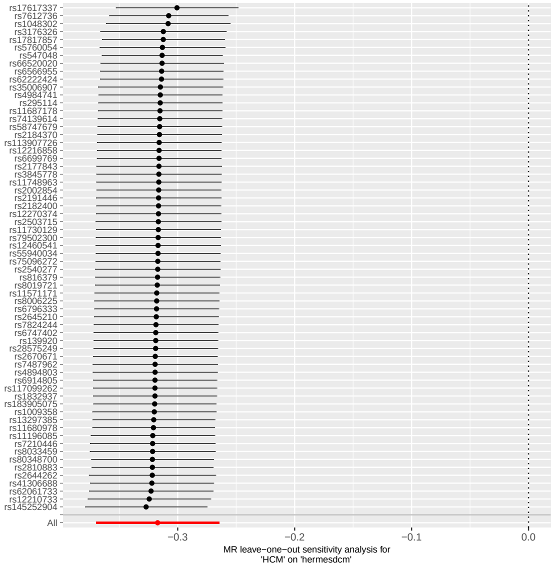

(c) Mendelian randomization single SNP forest plot for HCM as exposure on DCM.

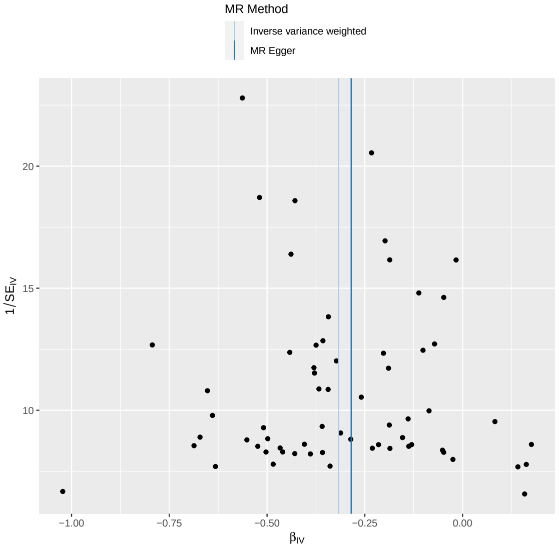

(d) Mendelian randomization leave one out plot for HCM as exposure on DCM.

Supplementary Fig. 9. Mendelian randomization analysis of HCM as exposure on DCM outcome. The plots show summary information on the analyses, performed as per the TwoSampleMR R package. The comparisons were completed using publicly available GWAS results (see Methods). Error bars represent standard error.

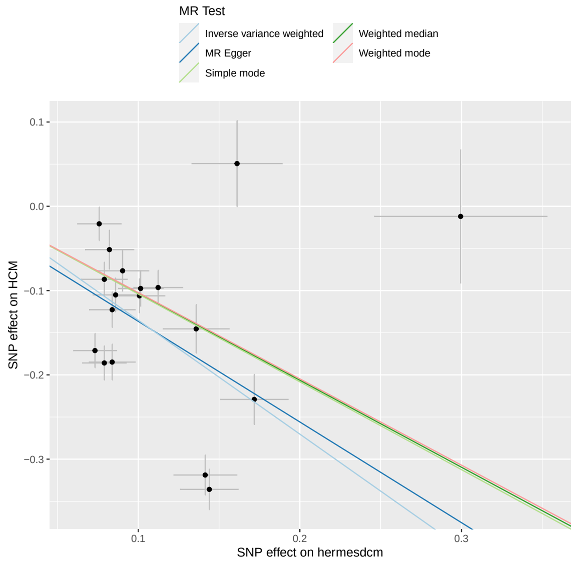

(a) Mendelian randomization scatter plot for DCM as exposure on HCM.

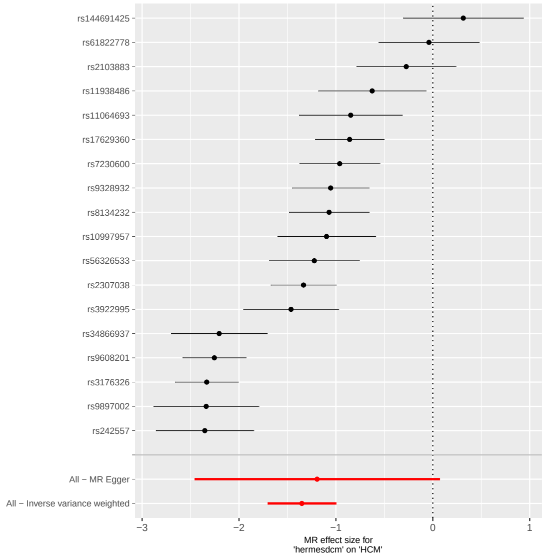

(b) Mendelian randomization single SNP funnel plot for DCM as exposure on HCM.

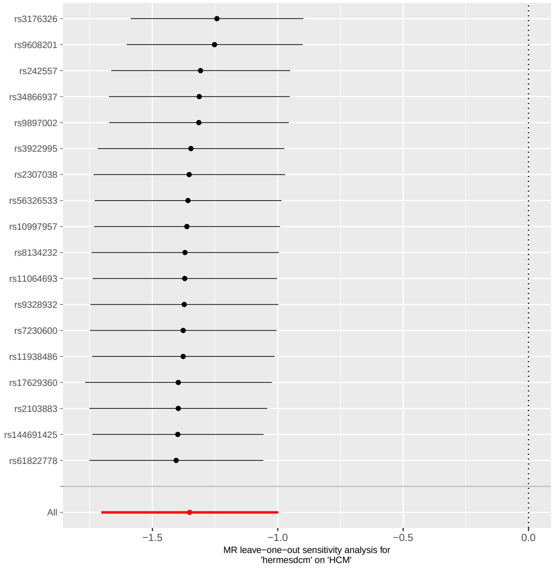

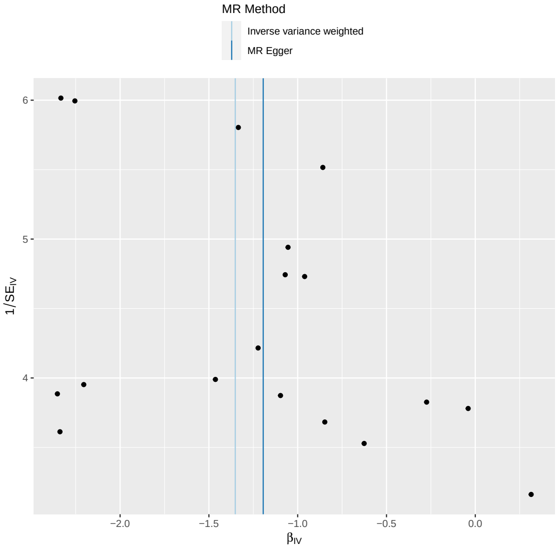

(c) Mendelian randomization single SNP forest plot for DCM as exposure on HCM. **(d)** Mendelian randomization leave one out plot for DCM as exposure on HCM.

Supplementary Fig. 10. Mendelian randomization analysis of DCM as exposure on HCM outcome. The plots show summary information on the analyses, performed as per the TwoSampleMR R package. The comparisons were completed using publicly available GWAS results (see Methods). Error bars represent standard error.

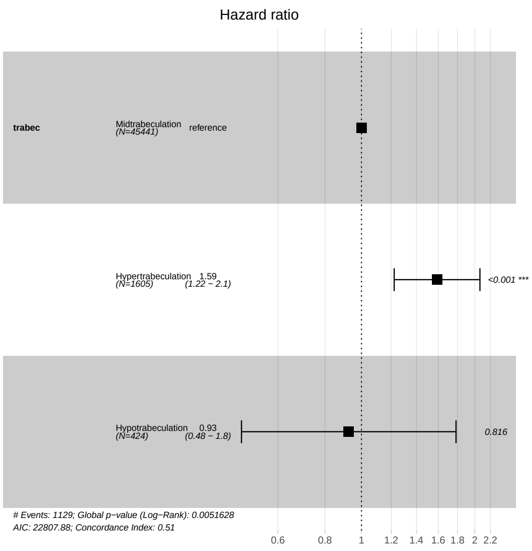

(a) 2 SD with heart failure.

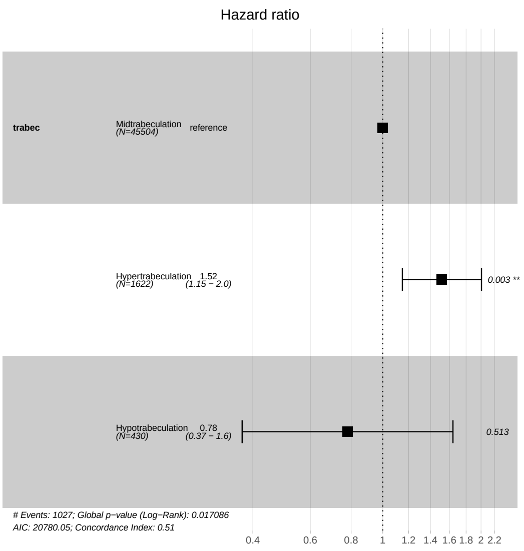

(b) 2 SD with mitral valve disease.

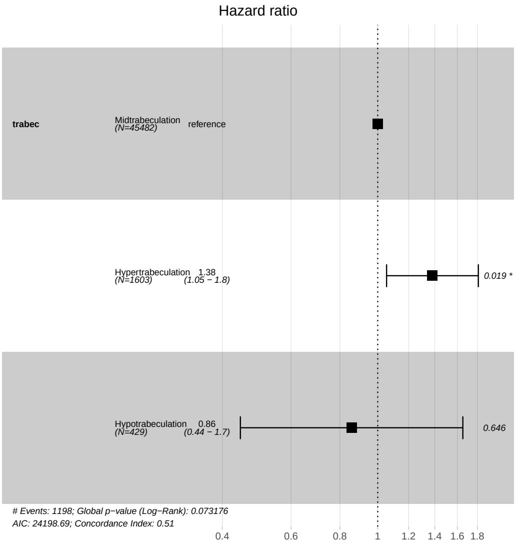

(c) 2 SD with bundle branch block.

Supplementary Fig. 11. Sensitivity analysis of the association of trabeculation outside 1.5 SD with clinical outcomes. a-c) by 2 SD (i.e., more extreme) for a) heart failure (hypertrabeculation P=0.00055), b) mitral valve disease, and c) bundle branch block. The mean coxfit linear predictors were plotted for trabeculation by group. Confidence intervals and log-rank p-values are depicted. CI, concordance index.

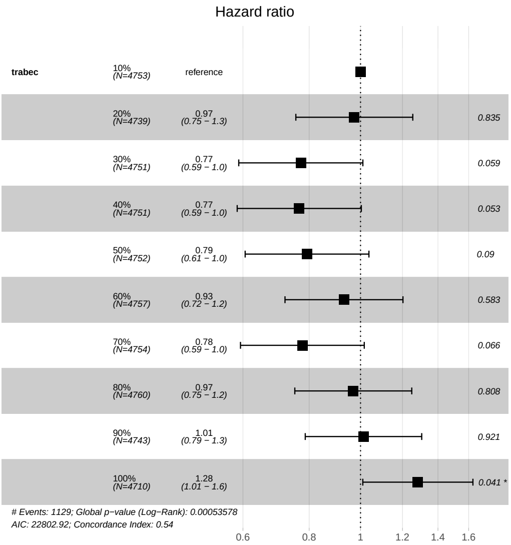

(a) Deciles with heart failure. Also presented in Extended Data Fig. 6.

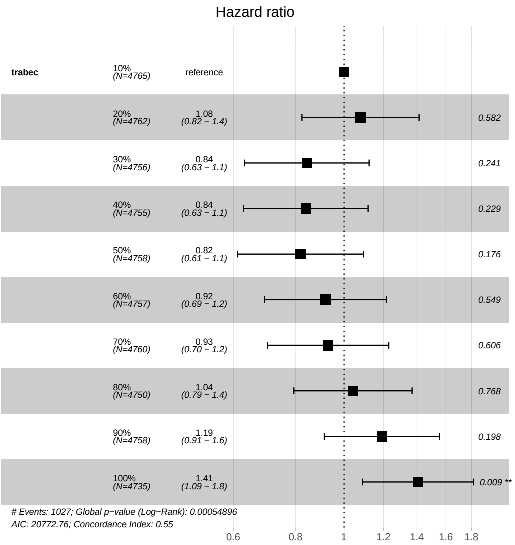

(b) Deciles with mitral valve disease.

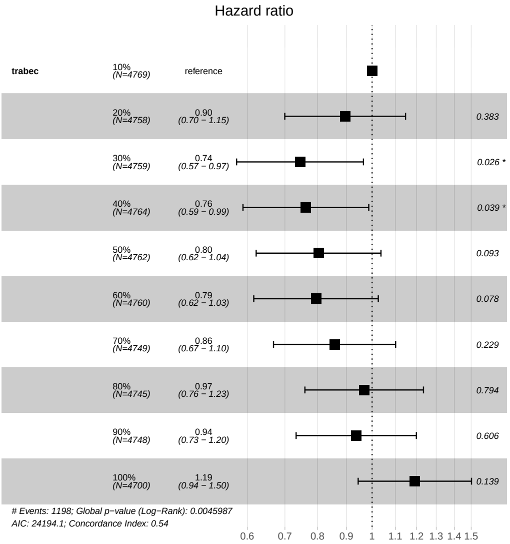

(c) Deciles with bundle branch block.

Supplementary Fig. 12. Sensitivity analysis of the association of trabeculation outside 1.5 SD with clinical outcomes. a-c) by deciles (limited statistical power) for a) heart failure, b) mitral valve disease, and c) bundle branch block. The mean coxfit linear predictors were plotted for trabeculation by group. Confidence intervals and log-rank p-values are depicted. CI, concordance index.

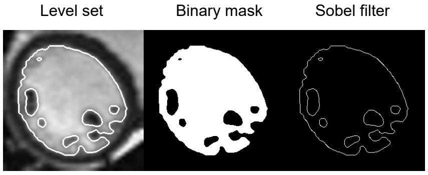

Supplementary Fig. 13. Trabecular analysis. Short axis image example with the level-set outline of the trabeculae, followed by conversion to a binary mask, on which a Sobel filter is applied to generate an outline for box-counting.
